# RESCUE: imputing dropout events in single-cell RNA-sequencing data

**DOI:** 10.1186/s12859-019-2977-0

**Published:** 2019-07-12

**Authors:** Sam Tracy, Guo-Cheng Yuan, Ruben Dries

**Affiliations:** 1 Department of Biostatistics, Harvard T.H. Chan School of Public Health, Boston, MA 02115 USA; 2 Department of Biostatistics and Computational Biology, Dana-Farber Cancer Institute, Boston, MA 02215 USA

**Keywords:** Dropout, Imputation, Bootstrap, Single-cell, RNA-seq

## Abstract

**Background:**

Single-cell RNA-sequencing technologies provide a powerful tool for systematic dissection of cellular heterogeneity. However, the prevalence of dropout events imposes complications during data analysis and, despite numerous efforts from the community, this challenge has yet to be solved.

**Results:**

Here we present a computational method, called RESCUE, to mitigate the dropout problem by imputing gene expression levels using information from other cells with similar patterns. Unlike existing methods, we use an ensemble-based approach to minimize the feature selection bias on imputation. By comparative analysis of simulated and real single-cell RNA-seq datasets, we show that RESCUE outperforms existing methods in terms of imputation accuracy which leads to more precise cell-type identification.

**Conclusions:**

Taken together, these results suggest that RESCUE is a useful tool for mitigating dropouts in single-cell RNA-seq data. RESCUE is implemented in R and available at https://github.com/seasamgo/rescue.

**Electronic supplementary material:**

The online version of this article (10.1186/s12859-019-2977-0) contains supplementary material, which is available to authorized users.

## Background

Single-cell RNA-seq (scRNAseq) analysis has been widely used to systematically characterize cellular heterogeneity within a tissue sample and offered new insights into development and diseases [1]. However, the quality of scRNAseq data is typically much lower than traditional bulk RNAseq. One of the most important drawbacks is dropout events, meaning that a gene which is expressed even at a relatively high level may be undetected due to technical limitations such as the inefficiency of reverse transcription [2]. Such errors are distinct from random sampling and can often lead to significant error in cell-type identification and downstream analyses [3].

Several computational methods have been recently developed to account for dropout events in scRNAseq data, either directly imputing under-detected expression values [4, 5], adjusting all values according to some model of the observed expression [6, 7] or implicitly accounting for missingness through the extraction of some underlying substructure [8]. Here we focus on directly imputing the missing information. In this context, imputation assumes that cells of a particular classification or type share identifiable gene expression patterns. Additionally, that missingness varies across cells within each type so that it is useful to borrow information from across cells with similar expression patterns, or cell neighbors. However, a challenge is that cell neighbor identification also relies on dropout-‘infected’ data, thus creating a chicken-and-the-egg problem. This problem has not been addressed in existing methods.

To overcome this challenge, we develop an algorithm called the **RE**covery of **S**ingle-**C**ell **U**nder-detected **E**xpression (RESCUE). The most important contribution of RESCUE is that the uncertainty of cell clustering is accounted for through a bootstrap procedure, thereby enhancing robustness. We apply RESCUE to simulated and biological data sets with simulated dropout and show that it accurately recovers gene expression values, improves cell-type identification and outperforms existing methods.

## Results

### Overview of the RESCUE method

**Fig. 1:**
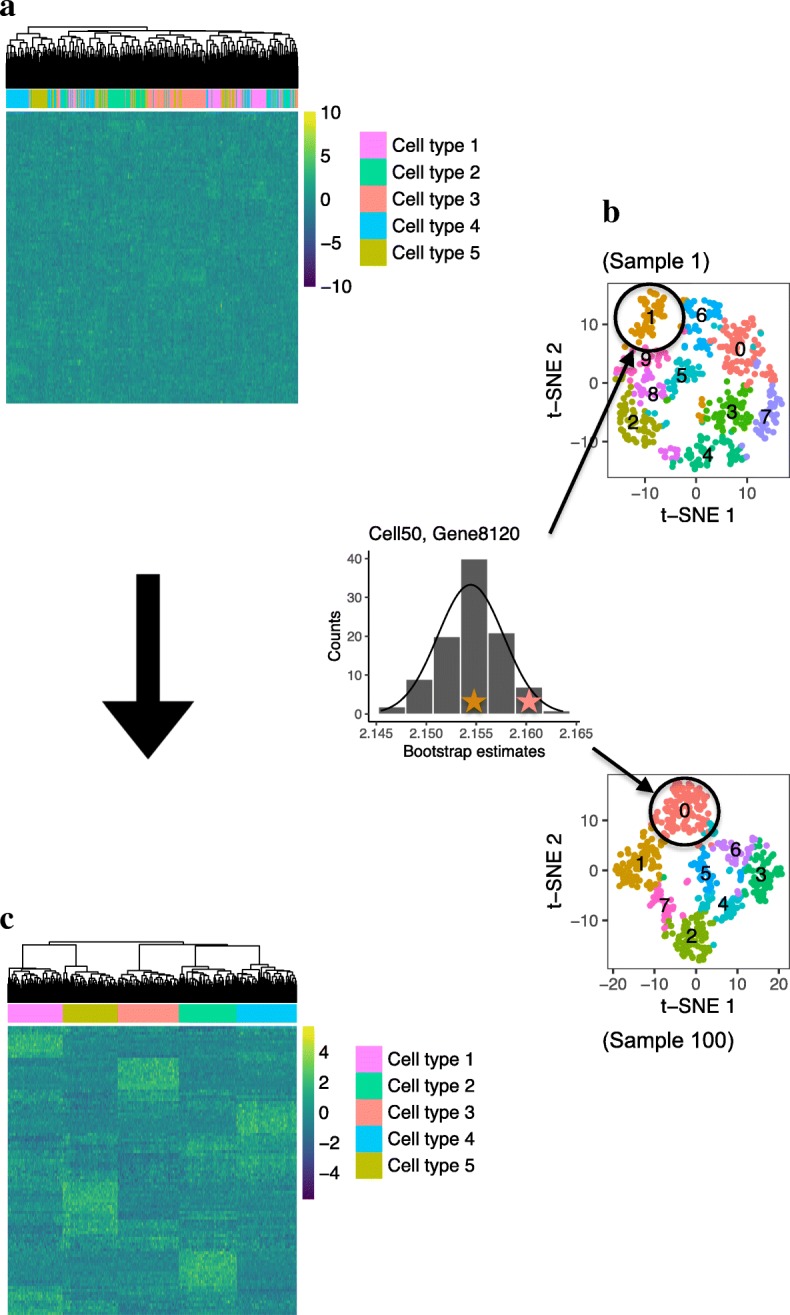
A motivation of the RESCUE imputation pipeline illustrated with a hypothetical example of simulated data. **a** Heatmap of a log-transformed normalized expression matrix with cell type clustering affected by dropout. **b** t-SNE visualizations of cell clusters determined with the principle components of many subsamples of informative genes, and a histogram showing the bootstrap distribution of the within-cluster non-zero gene expression means for one missing expression value in the data set. **c** Heatmap of the expression data after imputing zero values with a summary statistic of the bootstrap distributions

To motivate RESCUE, we note that cell-type clustering is typically restricted to a subset of informative genes, such as the most highly variable genes (HVGs) across all cells [9]. If there is bias in the expression patterns of these HVGs, then clustering will be affected. To illustrate this, we consider an idealized example of 500 cells containing five distinct cell types of near equal size. The introduction of dropout events distorts the pattern of gene expression and confounds clustering results by cell type (Fig. 1a). Our solution to this problem is to use a bootstrap procedure to generate many subsets of HVGs. Based on each subset of genes, we cluster cells based on the corresponding gene expression signatures and created an imputation estimate by within-cluster averaging (Fig. 1b). The final imputed data set provides an accurate representation of the cell types and their gene expression patterns (Fig. 1c).

Of note, this approach circumvents a number of limitations inherent to current imputation methods reviewed by Zhang and Zhang [10], as we’ve made no assumptions of the dropout generating mechanism or number of cell types and observed expression values are preserved.

More explicitly, given a normalized and log-transformed expression matrix, the RESCUE algorithm proceeds as follows. First, we consider the most informative features for determining cell neighbors. In this case, the most variable genes across all cells. We take a greedy approach and retain the top 1000 HVGs. The influence of any one group of genes is mitigated by repeatedly subsampling a proportion of HVGs with replacement, using the standard bootstrapping procedure [11] but with an additional clustering step for each estimator. Within each subsample, the gene expression data are standardized and reduced to their principal components to inform clustering. In principle, any single-cell clustering method [12] can be applied. As an example, here we use the shared nearest neighbors (SNN), which has been shown to be effective in numerous studies [13, 14]. As similar cells are assumed to share expression patterns, we calculate the average within-cluster expression for every gene in the data set as sample-specific imputations. In the end, the sample-specific imputation values are averaged for a final imputation. The mathematical details of the algorithm are described in the Methods section.

### RESCUE recovers under-detected expression in simulated data

As a ground truth is not generally known with experimental data, we first considered simulations for validation of RESCUE. Count data and dropout were simulated for a benchmark data set reflective of our hypothetical motivating example using generalized linear mixed models implemented by Splatter [15]. These data consisted of 500 cells having 10,000 genes and were composed of five distinct groups with equal probabilities of membership. Approximately 40% of observations had a true simulated count of 0 and approximately 30% of the overall transcripts counts experiencing additional dropout. To quantify the effect of dropout and imputation, the absolute count estimation error was evaluated relative to the simulated true counts. This measure is presented as the percent difference from the true counts over the data containing dropout so that 0% is best and greater than 100% indicates additional error. We used t-distributed stochastic neighbor embedding (t-SNE) [16] to visualize the data and determine the quality and separation of clusters by cell types. Additionally, we evaluated predicted cell type labels by computing their Shannon entropy, normalized mutual information (NMI), adjusted Rand Index (ARI), and Jaccard Index against their known cell type labels. The outcomes for these measures are presented as the percent improvement over the data containing dropout so that 100% is best and 0% is no improvement.

**Fig. 2:**
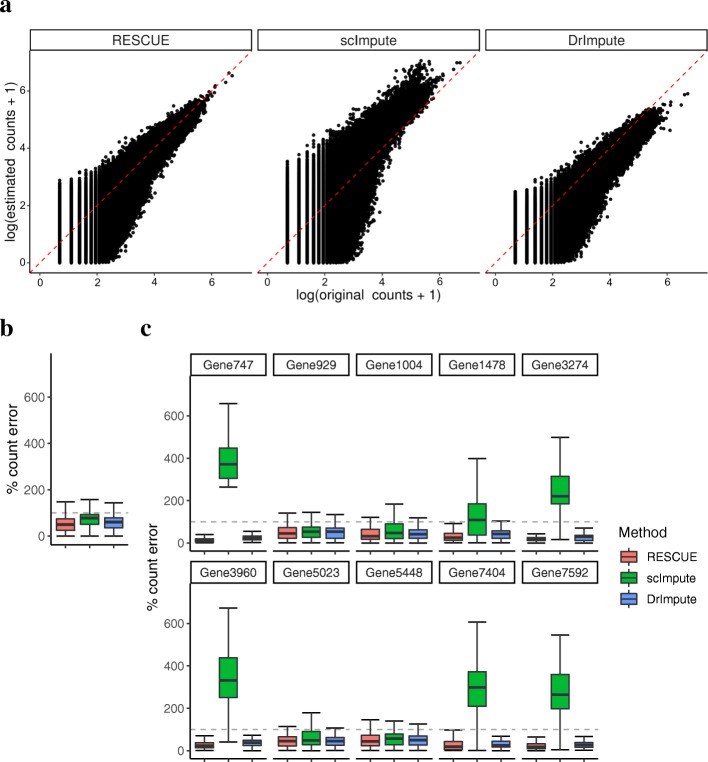
Estimation bias after imputing simulated data (Additional file 14: Table S1; Primary). **a** Scatter plots compare the true transcript counts (x-axis) to estimated counts (y-axis) for those lost to dropout. The red diagonal indicates unbiased estimation. **b** The percent absolute error for all missing counts. **c** The percent error for counts specific to the top ten marker genes across cell types. The dashed lines indicate 100% error, or no improvement over dropout

**Fig. 3:**
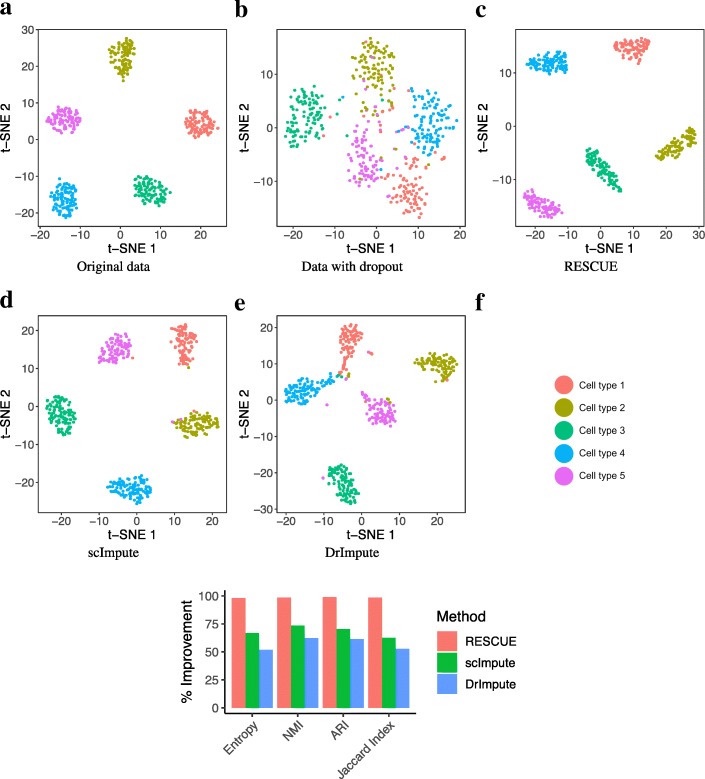
Data visualization and cell-type clustering before and after imputing simulated data (Additional file 14: Table S1; Primary). **a** t-SNE visualization of the original data labeled by cell type. **b** t-SNE after dropout **c** t-SNE after application of RESCUE. **d** t-SNE after application of scImpute. **e** t-SNE after application of DrImpute. **f** The percent improvement after imputation over the data containing dropout in similarity measures between known cell types and clustering results

Missing counts showed marked improvement (Fig. 2a) and RESCUE achieved a median reduction in total relative absolute error of 50% (Fig. 2b), indicating that our method can accurately recover the under-detected expression at a broad level. To ensure that missing expression values important to the classification of cell types were recovered, we considered the relative error for the top two most significantly differentially expressed marker genes for each cell type determined using the true counts (MAST [17] likelihood ratio test *p* <1*e* − 5; log-fold change >0.5). RESCUE achieved a median reduction in total relative absolute error of 50% (Fig. 2c). Additionally, RESCUE showed clear visual (Fig. 3a-c) and quantitative (Fig. 3f) improvement of cell-type classification. All five cell types were completely separated and clustering outcomes equivalent to the full data with a 0% difference from the true labels.

For comparison, we also imputed the dropout data with DrImpute [5] and scImpute [4], two recently developed methods designed to estimate under-detected expression values. Both methods reduced the relative absolute error (Fig. 2b) and DrImpute consistently reduced the relative absolute error across all 10 marker genes (Fig. 2c), but to a lesser degree than RESCUE. scImpute did not achieve the same reduction in error, instead having a noticeable increase in error for 6 of the 10 genes, possibly due to an overestimation of some counts (Fig. 2a). Both methods showed notable visual (Fig. 3d, e) and quantitative (Fig. 3f) improvement of clustering outcomes over the data set containing dropout, greater than 30% for DrImpute and greater than 90% for scImpute, but not to the same extent as RESCUE.

These outcomes were replicated in additional simulations (Additional file 1: Figure S1, Additional file 2: Figure S2, Additional file 3: Figure S3 and Additional file 4: Figure S4) that considered variations in cell group size, the number of cell types, degrees of differential expression, and the prevalence of dropout events outlined in Additional file 14: Table S1. Collectively, the simulations suggest that RESCUE is effective at recovering under-detected expression and outperforms existing methods in terms of estimation bias and clustering outcomes with regard to cell-type classification.

### RESCUE recovers differential expression across mouse cell types

To extend the application of RESCUE to a real data set where the underlying truth and mechanism are not fully known, we made use of the Mouse Cell Atlas (MCA) Microwell-seq data set [18]. Previous studies have identified 98 major cell types across 43 tissues [19]. We randomly selected four tissues — uterus, lung, pancreas and bladder — each of 1500 cells to test the performance of RESCUE. For each tissue, we only retained the cells that can be classified in a major cell-type for evaluation purposes. Since it is impossible to distinguish dropout events from biologically relevant low expression in this real dataset, we artificially introduced additional dropout events by using Splatter [15]. More than 10% of additional dropouts were introduced for each tissue. Genes having less than 10% of counts greater than zero within at least one cell type were removed. As a result, the data matrix for each tissue contained approximately 98% zero counts.

**Fig. 4:**
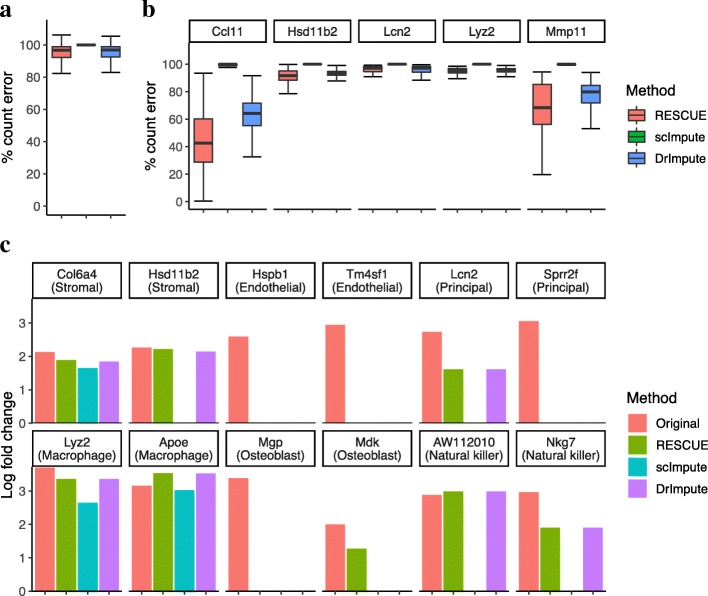
Estimation bias and recovery of differential expression after imputing the MCA uterus tissue data. **a** The percent absolute error for all missing counts. **b** The percent error for counts specific to top marker genes across cell types. Above 100% indicates no improvement over the data containing simulated dropout. **c** Log-fold changes in the two most differentially expressed marker genes for each cell type that went undetected after dropout

Missing counts showed a global median improvement of only 3% after imputing the uterus tissue data (Fig. 4a). However, RESCUE achieved a notable reduction of relative error across several of the most differentially expressed significant cell-type specific marker genes determined through a differential expression analysis (MAST [17] likelihood ratio test *p* <1*e* − 5; log-fold change >2) of the original counts (Fig. 4b). In particular, the *Ccl11* and *Mmp11* genes had a median reduction in error of 42 and 68%, respectively. This recovery of expression at a broad level and across marker genes was further replicated across the other three tissue types (Additional file 5: Figure S5, Additional file 6: Figure S6 and Additional file 7: Figure S7). We also evaluated the recovery of log-fold changes (LFCs) in gene expression for cell-type specific genes that went undetected in the data containing simulated dropout. RESCUE recovered 53 of the 77 significant genes in the uterus tissue (Additional file 15: Table S2), with six of these being the 2 most significant differentially expressed marker genes for each cell type (Fig. 4c). Similar results were achieved for the bladder, lung and uterus tissue data where LFC patterns were recaptured for a majority of each of the top two marker genes across cell types (Additional file 5: Figure S5, Additional file 6: Figure S6 and Additional file 7: Figure S7).

In contrast, other imputation methods achieved improvements in parts but not all of these elements. scImpute did not noticeably reduce count bias due to dropout events but recovered 100 marker genes across the cell types of each tissue (Additional file 15: Table S2). DrImpute had more similar results to RESCUE, reducing the overall relative error and error across marker genes, though not to the same degree. For example, the *Ccl11* and *Mmp11* genes had a median reduction in error of 64 and 80%, respectively (Fig. 4b). DrImpute also recovered an additional 5 marker genes in the lung tissue data (Additional file 15: Table S2) and the second most significant differentially expressed marker, *Wfdc2*, for urothelium cells in the bladder tissue, where RESCUE did not (Additional file 5: Figure S5c). However, RESCUE managed to recover several other markers in each tissue that were not detected after imputing with the other methods, including top markers *Mdk* (Fig. 4c), *H2 − Ab1* and *Myl9* (Additional file 5: Figure S5c), *Ms4a6c* (Additional file 6: Figure S6c) and *Gsn* (Additional file 7: Figure S7c). Together with the reduction in count bias, these results indicate that RESCUE can recover patterns of differential expression with regard to cell-type specific marker genes in the presence of heavy dropout.

### RESCUE improves cell-type classification of mouse cells

**Fig. 5:**
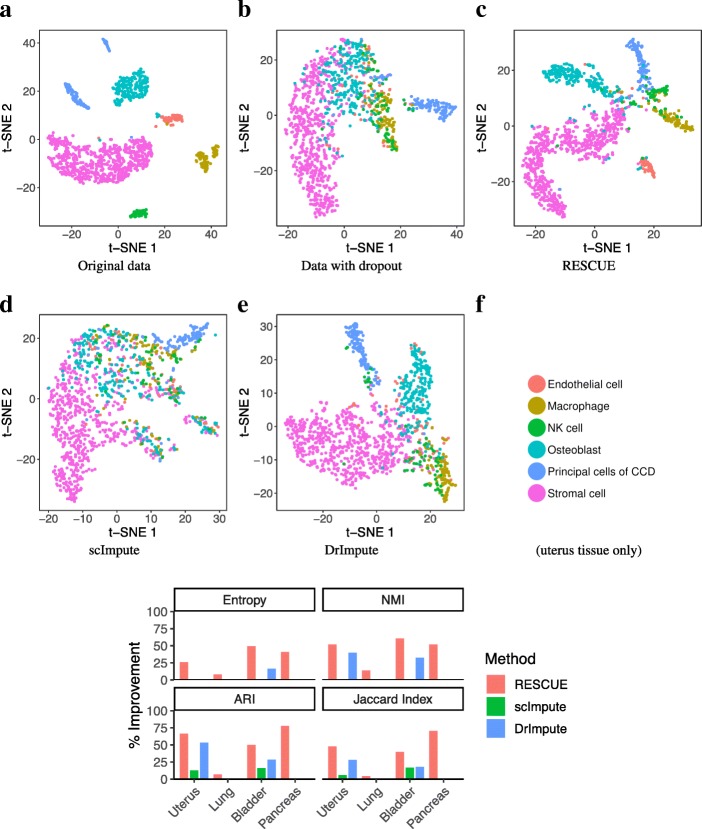
Data visualization and cell-type clustering before and after imputing the MCA data. **a** t-SNE visualization of the original uterus tissue data labeled by cell type. **b** t-SNE after dropout **c** t-SNE after application of RESCUE. **d** t-SNE after application of scImpute. **e** t-SNE after application of DrImpute. **f** The percent improvement after imputation over the data containing dropout in similarity measures between known cell types and clustering results for all four tissue types

To test whether RESCUE is useful for improving the accuracy of cell type identification, we overlaid the known cell-type annotation on t-SNE maps reconstructed from original, dropout, and imputed data (Fig. 5). RESCUE greatly enhanced the visual quality of the data clusters in the uterus tissue (Fig. 5a-c), clearly separating all six cell types. In particular, the endothelial cells and osteoblasts were indistinguishable from the other cells after dropout but visually distinct after imputation. A small number of cells were inseparable across cell types. However, this is seen in the original data and may be due to other sources of bias. RESCUE also improved clustering outcomes with regards to all considered measures (Fig. 5f). We compared estimated cell clusters with the cell-type labels identified using the full 60,000 cell data set in the original MCA study [19]. The relative entropy between these labels improved by 27%, NMI by 53%, ARI by 68%, and the Jaccard Index by 49%. To test if the improvement is robust, we repeated the analysis for three additional tissues: bladder (Additional file 5: Figure S5), lung (Additional file 6: Figure S6) and pancreas tissues (Additional file 7: Figure S7). In all cases, we observed varying degree of improvement of RESCUE compared to existing methods.

Some of the more similar cell types were inseparable after additional dropout. For example, the dendritic cells and monocytes in the lung tissue are partly distinct in the original data but cluster together and remain indistinguishable after imputation (Additional file 9: Figure S9c). This could be due to a complete loss of some information distinguishing these cells, as differential expression for top dendritic cell markers was not recovered (Additional file 6: Figure S6c). However, we see this again with the dendritic cells and macrophages in the bladder tissue (Additional file 8: Figure S8c). These three immune cell types are known to greatly overlap in both functional characteristics and patterns of gene expression [20], confounding their separate classification. Thus, this event may simply be confined to similarly expressing immune cells in the presence of other dissimilar cell types. We do observe that the immune cells of both tissues become visibly distinct from other cell types with imputation, indicating a meaningful improvement in overall cell-type classification.

Other methods underperformed RESCUE in these outcomes. scImpute increased the similarity indexes for the uterus and bladder tissue data but did not reduce entropy or increase the NMI between the known cell labels or improve clustering outcomes across the other tissue types (Fig. 5f)*.* Visualization of the data with t-SNE did not improve either (Fig. 5d, Additional file 8: Figure S8, Additional file 9: Figure S9 and Additional file 10: Figure S10). In contrast, DrImpute showed visible improvement across all measures predicted clustering quality for the uterus and bladder tissue data but to a lesser degree than RESCUE; this was not seen with the pancreas and lung tissue data (Fig. 5f) and was not fully apparent in visualization of the data with t-SNE (Fig. 5e, Additional file 8: Figure S8, Additional file 9: Figure S9 and Additional file 10: Figure S10). We conclude that RESCUE improves clustering outcomes and the accuracy of cell-type classification, while outperforming other existing methods in the presence of dropout.

## Discussion

Single-cell experiments and analyses have greatly improved over the last decade and are now considered an essential component in many research areas. However, their focus has primarily been at the transcriptome level, which is only one of many regulatory layers that explains single-cell heterogeneity. Recently, additional high-throughput single-cell sequencing protocols have been developed for analyzing patterns in DNA methylation and chromatin accessibility, such as the single-cell assay for transposase-accessible chromatin (ATAC-seq) [21]. These data are unique to scRNA-seq data but present similar challenges due to high amounts of background noise and low read-coverage [22]. The RESCUE method may not be directly applicable to these other data but, given its simplicity and straightforward approach, we place interest in future extensions.

## Conclusions

The identification of cell types is at the core of scRNAseq data analysis but confounded by high rates of under-detected expression that bias informative patterns of gene expression. RESCUE effectively recovered the information lost to these dropout events in both simulations and publicly available data with additional simulated dropout. Count error and feature selection bias were significantly reduced and differential expression patterns important to cell-type classification were recovered, significantly improving downstream cell-type clustering. This was achieved through two important additions to the literature. First, a solution to the inter-dependency of cell-type classification and estimation of gene expression by subsampling informative genes. Second, retaining the single-cell nature of the data without strict model assumptions by applying the bootstrap across all possible clustering outcomes. To improve computation time RESCUE optionally implements the bootstrap iterations in parallel, with a reduction in total time by up to half when using 10 cores (Additional file 11: Figure S11). Taken together with the above, we believe that RESCUE can be a useful addition to the current and developing toolsets used in the analysis of single-cell data.

## Methods

### Simulating single-cell RNA-sequencing data

Simulated data were generated using Splatter. Splatter implements a gamma-Poisson hierarchical model, an extended reparameterization of the common negative binomial model. Briefly, gene expression means are sampled from a gamma distribution and subsequent cell counts from a Poisson distribution [15]. Alone, this model would ignore many of the unique characteristics of scRNA-seq data, such as outlier genes and zero-inflation. These are accounted for by sampling additional parameters from a variety of statistical distributions that are then utilized throughout the hierarchical structure of the Splatter model. We considered three scenarios outlined in Additional file 14: Table S1, with remaining parameters kept at their default values. If any genes were to have zero counts across all cells, we removed them from that data set before imputation [23, 24].

### Mouse cell atlas data and processing

We obtained the Mouse Cell Atlas (MCA) data set of 60,000 single cells from the Gene Expression Omnibus under accession code GSE108097 [18]. Our selected 4-tissue subset was filtered by cell types to those having at least 50 cells present in each data set, with this threshold being lowered to 25 cells for the bladder tissue in order to capture more cell types. In this way, we reduced bias in the final clustering analysis due simply to rare cell types. We also filtered genes with a very low detection threshold across the remaining cells (<10 % nonzero counts within every remaining cell type). Both the simulated and sequenced data were processed with the *Seurat* pipeline implemented in R [25] using default parameters for quality control, normalization (log-transformed counts-per-million), UMI regression of the MCA data, and scaling (z-score).

### Generating dropout events

The Splatter model generates dropout in a manner in consonance with the findings of Hicks, Townes [3]. Specifically, dropout probabilities are defined by use of the logistic function $f(x)={\left(1+{e}^{-a\left(x-{x}_0\right)}\right)}^{-1}$ fit between the log means of the normalized counts and the proportion of under-detected counts. Dropout is then generated with these probabilities and counts replaced by zero as such events occur. These methods are implemented in the R package Splatter [15]. We fixed the dropout.midpoint location parameter *x*_0_ = 0 for all data sets. Dropout for the simulated data was generated with the parameters given in Additional file 14: Table S1. Data specific parameters for the MCA data were estimated using the splatEstimate function. The dropout.shape scale parameter was fixed at *a* =  − 1 and the model parameter dropout.type to ‘experiment’. We then generated an index of dropout events using the splatSimulate function with cell type probability parameter group.prob. set to the proportion of known cell types. Counts sampled in this way were changed to zero. This resulted in more than 10% additional dropout across each of the MCA tissues we evaluated.

### Mathematical details of RESCUE

RESCUE takes as input a normalized and log-transformed gene expression matrix. The algorithm then proceeds as follows:

1. HVGs were determined with the FindVariableGenes function in the R package Seurat [25]. Seurat separates the genes by their average expression into twenty bins, then thresholds and ranks genes within each bin by the ratio of their variance and mean. We filtered genes to have an average non-zero log-transformed expression and took the top-ranked 1000 remaining genes.
2. Simulations across multiple proportions *p* of HVGs suggested a window in which the variation of informative clustering outcomes was optimal (Additional file 12: Figure S12). We fixed *p* at a conservative 0.6 within this window to capture a simple majority of HVGs and ensure that the expression pattern of each subsample was representative of cell type but flexible across all HVGs.
3. Cell clusters are also determined via the Seurat package with the FindClusters function. This implementation of SNN borrows heavily from Levine, Simonds [14] and first draws a KNN graph over the Euclidean distance of informative principal components. We determined the number of principal components by examining elbow plots computed with the full set of 1000 HVGs and these may be increased as desired. The graph edge weights are refined by the Jaccard distance between local neighborhoods and groups of highly connected cells are partitioned by the Louvain modularity optimization method proposed by Blondel, Guillaume [26]. This requires a resolution parameter as input to adjust the granularity of the community partitions; greater than 1 induces more clusters, while less than 1 induces fewer clusters. We kept this parameter at a moderate value of 0.9, the original authors suggested best results for 0.6–1.2, but we experienced little variation in results across this window and it may be increased for large data sets where a greater number of unique cell types are expected.
4. Expression averages are calculated for each cluster.
5. Steps 2–4 are performed *N* times to extrapolate the distribution of expression averages across all possible cell neighbors. We fixed *N* at 100 to ensure consistency of the bootstrap after evaluating these distributions under simulation.
6. Take *c*_*i*_ to be a series of these estimated similar cell cluster identities assigned to some cell *c* with cluster size ${n}_{c_i}$ and for *i* = 1, …, *N*. Take some gene *g* having cluster-specific expression vectors ${x}_{g{c}_i}$ for *i* = 1, …, *N*, and denote its cluster-specific expression mean by *θ*_*gc*_. We define the estimated expression averages ${\overline{x}}_{g{c}_i}={n}_{c_i}^{-1}\cdotp {\sum}_j{x}_{g{c}_i,j}$ for $j=1,\dots, {n}_{c_i}$. Then, statistics computed with the estimator defined by

$$ {\hat{\theta}}_{gc}=\left\{\sum \limits_{i=1}^N{n}_{c_i}\bullet {\overline{x}}_{g{c}_i}\right\}\bullet {\left\{\sum \limits_{i=1}^N{n}_{c_i}\right\}}^{-1} $$

are the bootstrapped mean expression estimates of *θ*_*gc*_ for gene *g* in cell *c*. Zero counts are imputed with their respective estimates and the algorithm ends.

### Analysis with scImpute and DrImpute

scImpute initially clusters similar cells with KMeans applied to a spectral decomposition of the data [27] to reduce the computational effort of fitting a separate generalized linear mixed model to every sample, which takes as input the expected number of cell states [4]. scImpute performed better without informing the clustering algorithm and so we fixed the initial clustering parameter *ks* at 1. The authors state that this is fine as the method chooses similar cells with a model-based approach at a later step. Each data set was imputed before processing, as the method takes counts as input.

DrImpute implements multiple applications of KMeans clustering and correlation distances, suggesting a range of numbers of clusters for the applications of KMeans that are at least as large as the number of expected clusters [5] (the default is 10:15). Let *k* be the number of known cell types. We fixed the range of clusters for DrImpute to be {*k*, …, *k* + 5}. All other parameters were fixed at their default values.

### Evaluation of clustering outcomes and marker genes

Principal component analysis, SNN clustering and t-SNE visualization were implemented using The R package Seurat [25]. The entire filtered set of genes present in the data containing dropout was used for all evaluations. We measured count bias by retaining cell library sizes before imputation and applying an inverse function of the log-transform normalization *g*^−1^(*x*) = {exp(*x*) − 1} × 10^−4^ × *library* _ *size*. Log-fold changes and marker genes were determined through a differential expression analysis of the original filtered data with known cell-type labels using the FindMarkers function in the R package Seurat and MAST [17], a GLM method developed specifically for scRNAseq data that models the cell detection rate as a covariate. Genes were filtered by the magnitude of their LFC (>2.0 for the MCA data, >0.5 for the simulated data) and sorted by significance (likelihood ratio test *p* <1*e* − 5). A subset of the most significantly expressed marker genes, or top markers, were selected from each cell type in the original data set if they also went undetected in a subsequent analysis applied to the data set containing dropout. Similarity measures for predicted cell types were computed with the external_validation function in the R package ClusterR [28].

SNN does not predict a fixed number of clusters, instead producing a final number of clusters as a product of the optimal community partitions. Yet most measures of clustering quality are sensitive to variations in the number of unique clusters. Thus, it was necessary to reduce larger numbers of predicted clusters to the number of unique cell types for a quantitative evaluation of similarity to cell type labels. This was achieved by merging predicted clusters with average-linkage of the Euclidean distance across the same number of principal components used to inform the SNN clustering. The need for this is seen in the MCA bladder tissue data set, where the initial predicted clusters from the original data seem to poorly match cell types according to the plotted similarity measures (Additional file 13: Figure S13c). However, the original data is quite clearly accurate according to the t-SNE plots (Additional file 13: Figure S13a) when contrasted against the known cell labels (Additional file 8: Figure S8a).

## Additional files

Additional file 1: **Figure S1.** Estimation bias after imputing simulated data (Additional file 14: Table S1; Scenario 2). (a) . Scatter plots compare the true transcript counts (x-axis) to estimated counts (y-axis) for those lost to dropout. The red diagonal indicates unbiased estimation. (b) The percent absolute error for all missing counts. (c) The percent error for counts specific to the top ten marker genes across cell types. The dashed lines indicate 100% error, or no improvement over dropout. (PDF 1104 kb)

Additional file 2: **Figure S2.** Data visualization before and after imputing simulated data (Additional file 14: Table S1; Scenario 2). (a) t-SNE visualization of the original data labeled by cell type. (b) t-SNE after dropout (c) t-SNE after application of RESCUE. (d) t-SNE after application of scImpute. (e) t-SNE after application of DrImpute. (f) The percent improvement after imputation over the data containing dropout in similarity measures between known cell types and clustering results. (PDF 481 kb)

Additional file 3: **Figure S3.** Estimation bias after imputing simulated data (Additional file 14: Table S1; Scenario 3). (a) . Scatter plots compare the true transcript counts (x-axis) to estimated counts (y-axis) for those lost to dropout. The red diagonal indicates unbiased estimation. (b) The percent absolute error for all missing counts. (c) The percent error for counts specific to the top ten marker genes across cell types. The dashed lines indicate 100% error, or no improvement over dropout. (PDF 1131 kb)

Additional file 4: **Figure S4.** Data visualization before and after imputing simulated data (Additional file 14: Table S1; Scenario 3). (a) t-SNE visualization of the original data labeled by cell type. (b) t-SNE after dropout (c) t-SNE after application of RESCUE. (d) t-SNE after application of scImpute. (e) t-SNE after application of DrImpute. (f) The percent improvement after imputation over the data containing dropout in similarity measures between known cell types and clustering results. (PDF 483 kb)

Additional file 5: **Figure S5.** Estimation bias after imputing the MCA bladder tissue data. (a) The percent absolute error for all missing counts. (b) The percent error for counts specific to top marker genes across cell types. Above 100% indicates no improvement over the data containing simulated dropout. (c) Log-fold changes in the two most differentially expressed marker genes for each cell type that went undetected after dropout. (PDF 67 kb)

Additional file 6: **Figure S6.** Estimation bias after imputing the MCA lung tissue data. (a) The percent absolute error for all missing counts. (b) The percent error for counts specific to top marker genes across cell types. Above 100% indicates no improvement over the data containing simulated dropout. (c) Log-fold changes in the two most differentially expressed marker genes for each cell type that went undetected after dropout. (PDF 70 kb)

Additional file 7: **Figure S7.** Estimation bias after imputing the MCA pancreas tissue data. (a) The percent absolute error for all missing counts. (b) The percent error for counts specific to top marker genes across cell types. Above 100% indicates no improvement over the data containing simulated dropout. (c) Log-fold changes in the two most differentially expressed marker genes for each cell type that went undetected after dropout. (PDF 62 kb)

Additional file 8: **Figure S8.** Data visualization before and after imputing the MCA bladder tissue data. (a) t-SNE visualization of the original data labeled by cell type. (b) t-SNE after dropout (c) t-SNE after application of RESCUE. (d) t-SNE after application of scImpute. (e) t-SNE after application of DrImpute. (PDF 966 kb)

Additional file 9: **Figure S9.** Data visualization before and after imputing the MCA lung tissue data. (a) t-SNE visualization of the original data labeled by cell type. (b) t-SNE after dropout (c) t-SNE after application of RESCUE. (d) t-SNE after application of scImpute. (e) t-SNE after application of DrImpute. (PDF 888 kb)

Additional file 10: **Figure S10.** Data visualization before and after imputing the MCA pancreas tissue data. (a) t-SNE visualization of the original data labeled by cell type. (b) t-SNE after dropout (c) t-SNE after application of RESCUE. (d) t-SNE after application of scImpute. (e) t-SNE after application of DrImpute. (PDF 917 kb)

Additional file 11: **Figure S11.** Minutes of the RESCUE computation against sample size in Splatter simulations on the natural log-scale. (PDF 44 kb)

Additional file 12: **Figure S12.** Similarity measures between imputed and original data with different proportions *p* of subsampled genes in the first simulation scenario and the dropout rate parameter to − 0.25 in order to encourage the need for subsampling HVGs. (PDF 40 kb)

Additional file 13: **Figure S13.** Data visualization and clustering results before and after dropout in the MCA bladder tissue. (a) t-SNE visualization of the original uterus tissue data labeled by estimated clusters. (b) t-SNE after dropout. (c). (PDF 272 kb)

Additional file 14: **Table S1.** Splatter simulation parameters. (DOCX 14 kb)

Additional file 15: **Table S2.** Significant differentially expressed genes. (DOCX 14 kb)

## Data Availability

The Mouse Cell Atlas data set is available from the Gene Expression Omnibus under accession code GSE108097 [18]. The generated data are available from the corresponding author on reasonable request.

## Abbreviations

ARI: Adjusted Rand index
ATAC-seq: Assay for transposase-accessible chromatin sequencing
HVG: Highly variable gene
MAST: Model-based analysis of single-cell transcriptomics
MCA: Mouse Cell Atlas
NMI: Normalized mutual information
scRNAseq: Single-cell RNA sequencing
SNN: Shared nearest neighbors
t-SNE: t-Distributed stochastic neighbor embedding
