# Supplementary figures and images for: RESCUE: imputing dropout events in single-cell RNA-sequencing data
Source: BMC Bioinformatics. 2019 Jul 12;20:388. doi: 10.1186/s12859-019-2977-0 (PMC6624880; doi:10.1186/s12859-019-2977-0)

**Fig. S1****a**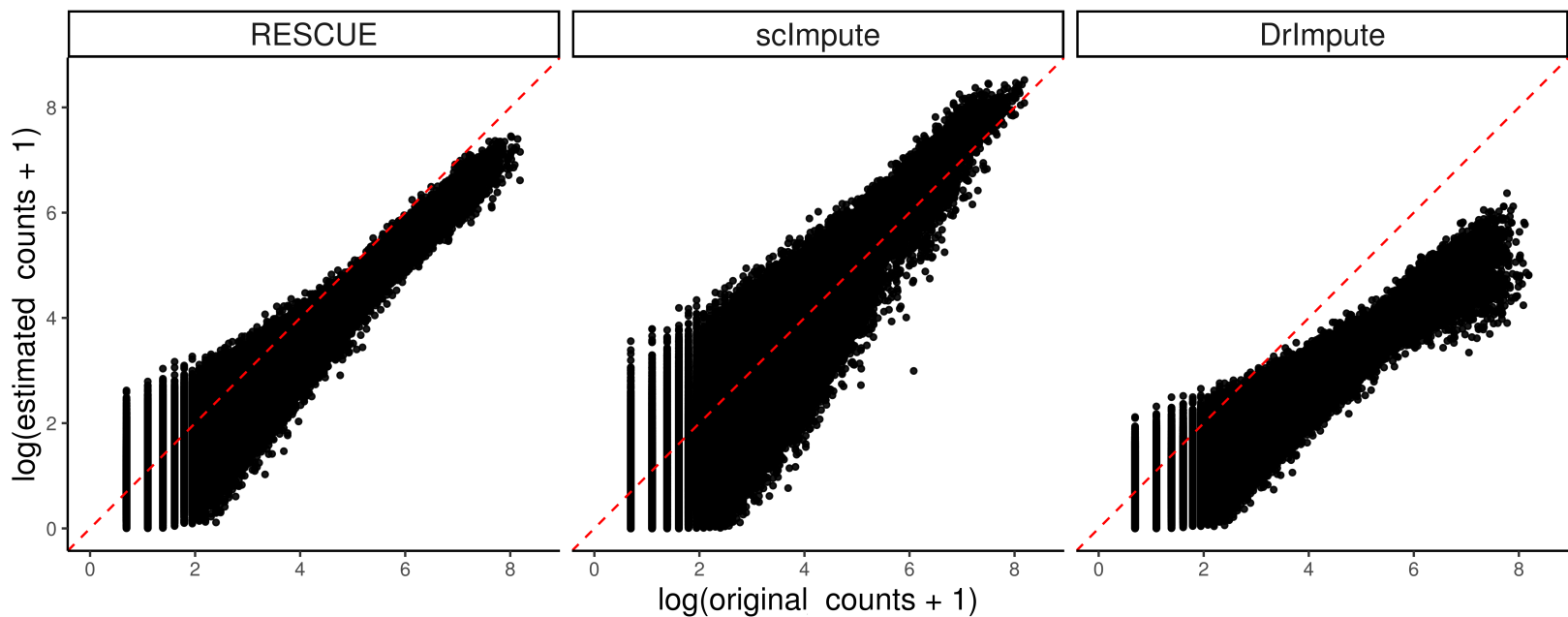**b**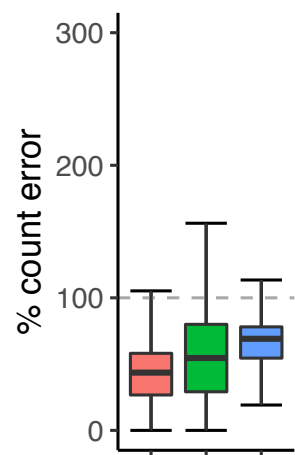**c**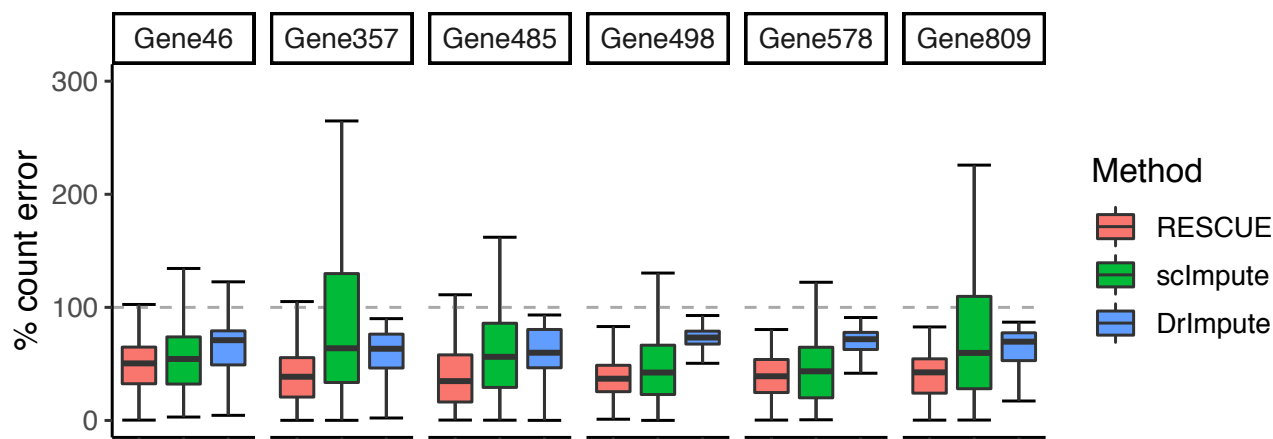

Supplement: Supplementary file 1 — Figure S1. Estimation bias after imputing simulated data (Additional file 14: Table S1; Scenario 2). (a) . Scatter plots compare the true transcript counts (x-axis) to estimated counts (y-axis) for those lost to dropout. The red diagonal indicates unbiased estimation. (b) The percent absolute error for all missing counts. (c) The percent error for counts specific to the top ten marker genes across cell types. The dashed lines indicate 100% error, or no improvement over dropout. (PDF 1104 kb) [file 12859_2019_2977_MOESM1_ESM.pdf]

**Fig. S2**

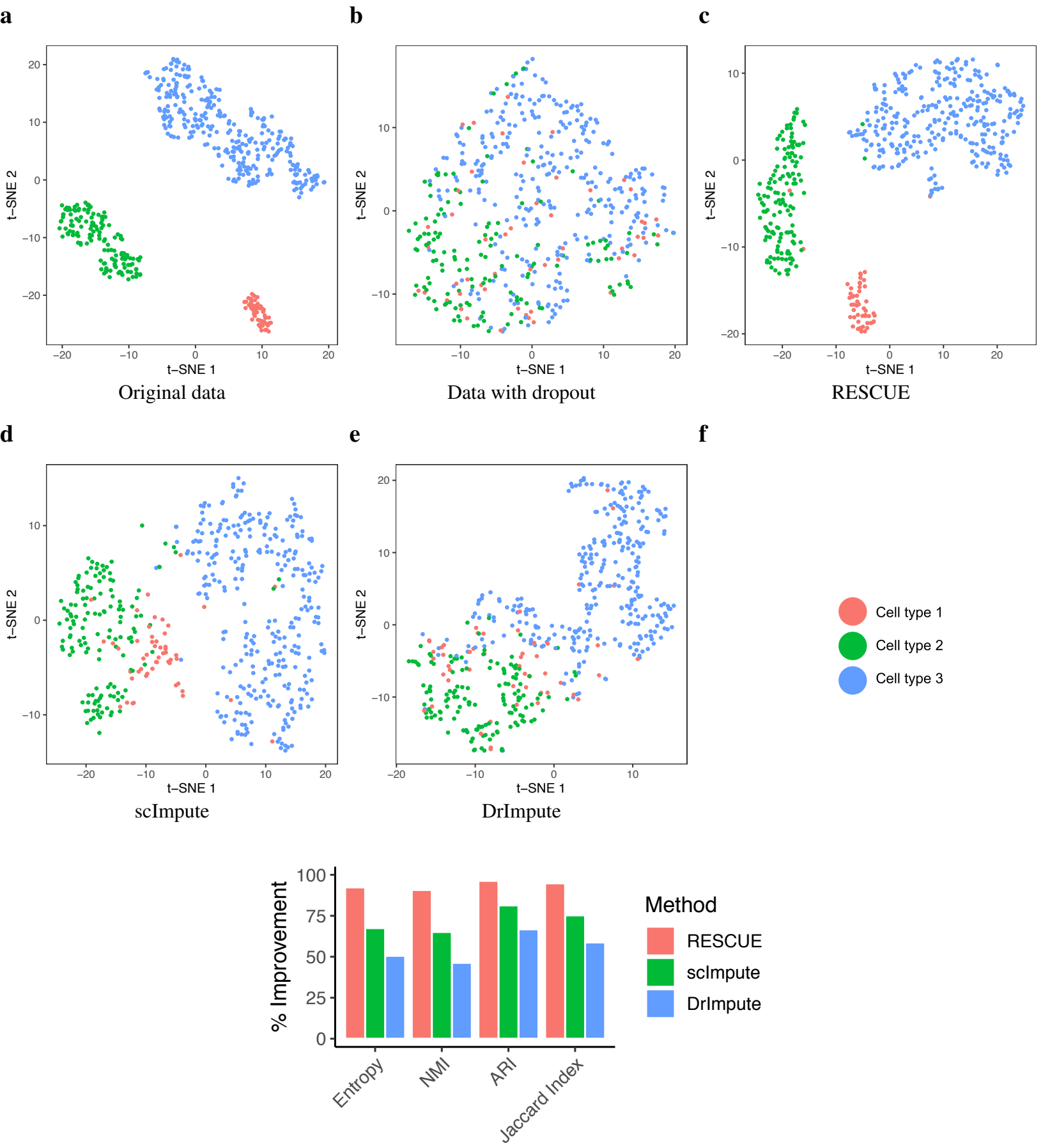

Supplement: Supplementary file 2 — Figure S2. Data visualization before and after imputing simulated data (Additional file 14: Table S1; Scenario 2). (a) t-SNE visualization of the original data labeled by cell type. (b) t-SNE after dropout (c) t-SNE after application of RESCUE. (d) t-SNE after application of scImpute. (e) t-SNE after application of DrImpute. (f) The percent improvement after imputation over the data containing dropout in similarity measures between known cell types and clustering results. (PDF 481 kb) [file 12859_2019_2977_MOESM2_ESM.pdf]

**Fig. S3****a**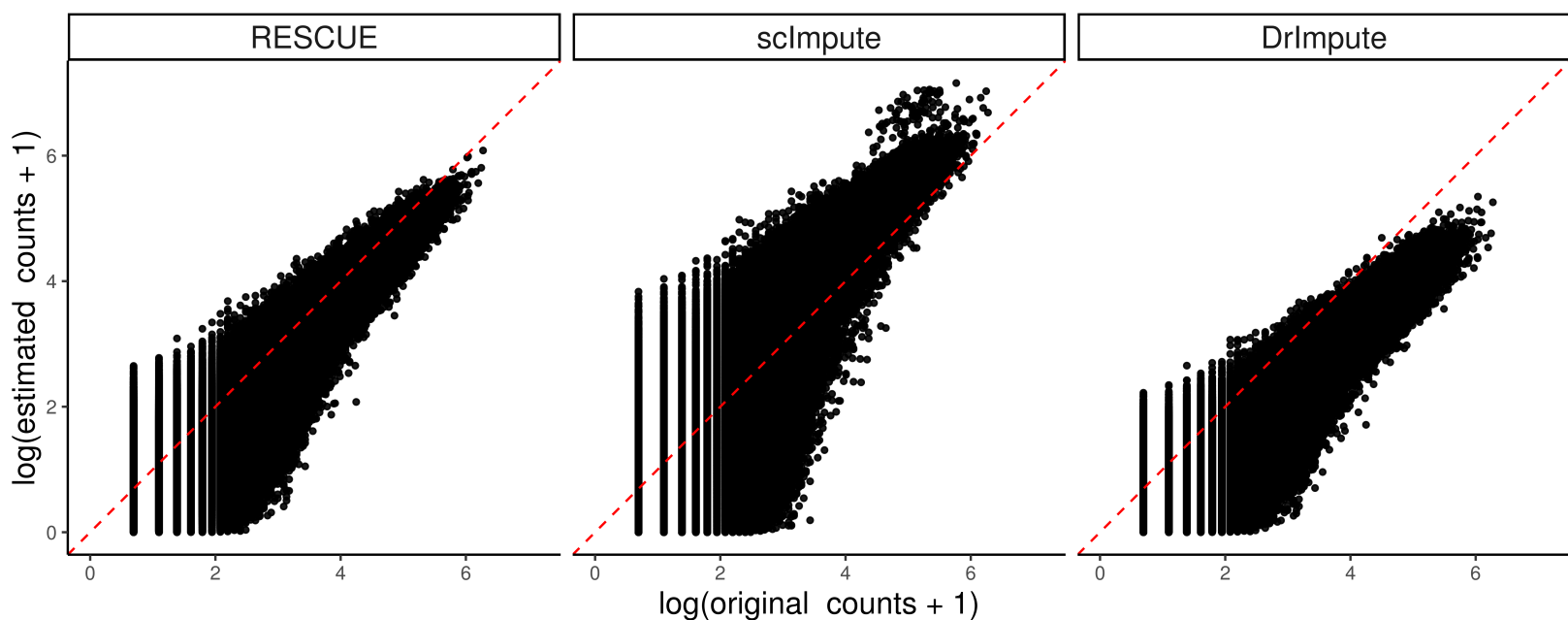**b**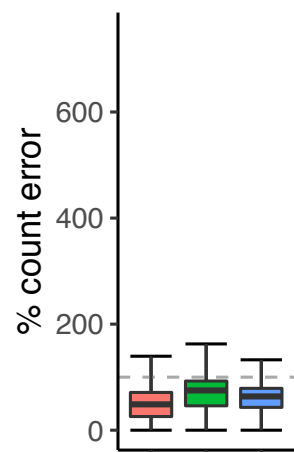**c**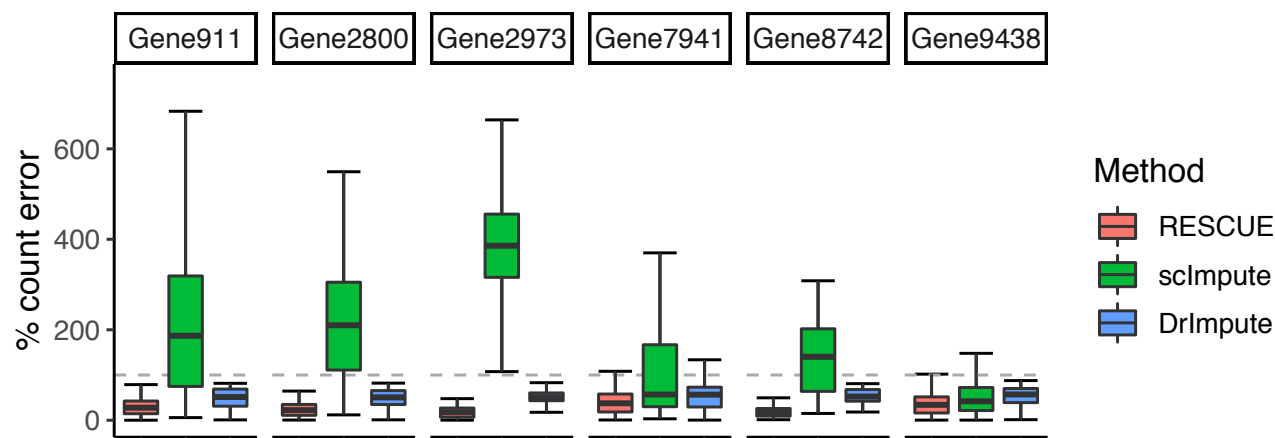

Supplement: Supplementary file 3 — Figure S3. Estimation bias after imputing simulated data (Additional file 14: Table S1; Scenario 3). (a) . Scatter plots compare the true transcript counts (x-axis) to estimated counts (y-axis) for those lost to dropout. The red diagonal indicates unbiased estimation. (b) The percent absolute error for all missing counts. (c) The percent error for counts specific to the top ten marker genes across cell types. The dashed lines indicate 100% error, or no improvement over dropout. (PDF 1131 kb) [file 12859_2019_2977_MOESM3_ESM.pdf]

**Fig. S4**

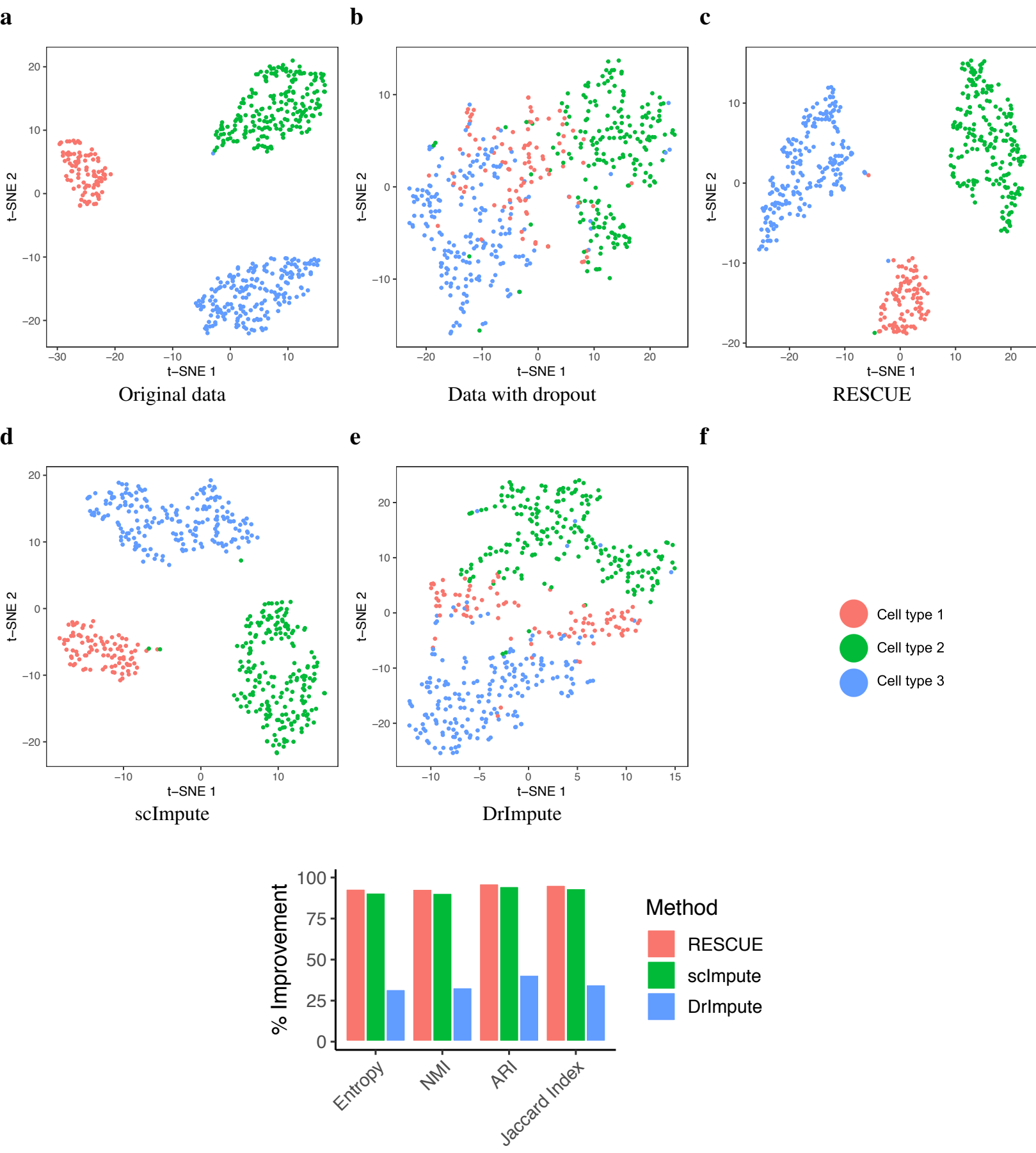

Supplement: Supplementary file 4 — Figure S4. Data visualization before and after imputing simulated data (Additional file 14: Table S1; Scenario 3). (a) t-SNE visualization of the original data labeled by cell type. (b) t-SNE after dropout (c) t-SNE after application of RESCUE. (d) t-SNE after application of scImpute. (e) t-SNE after application of DrImpute. (f) The percent improvement after imputation over the data containing dropout in similarity measures between known cell types and clustering results. (PDF 483 kb) [file 12859_2019_2977_MOESM4_ESM.pdf]

**Fig. S5****a**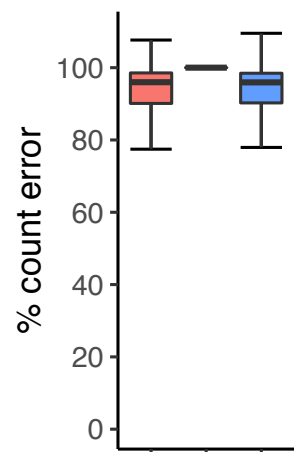**b**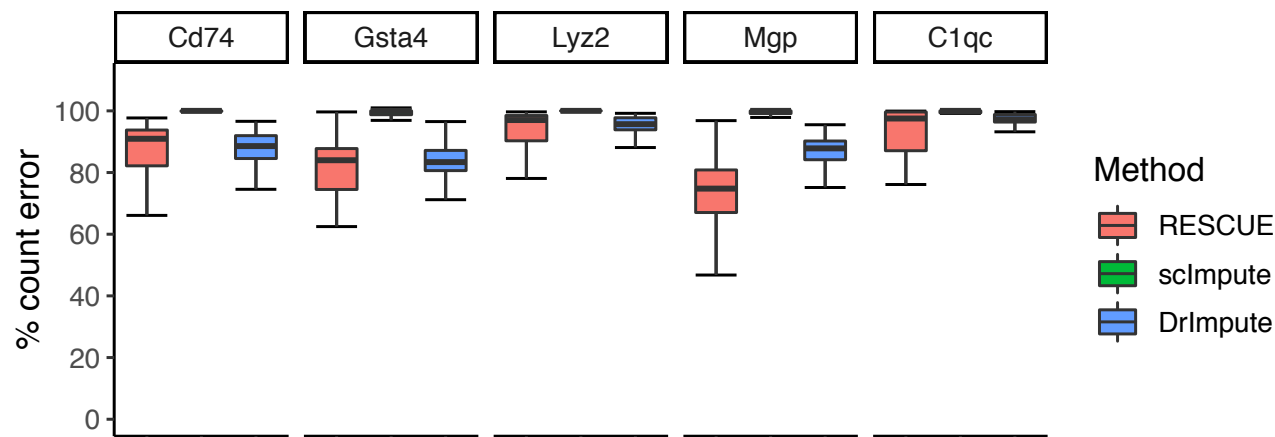**c**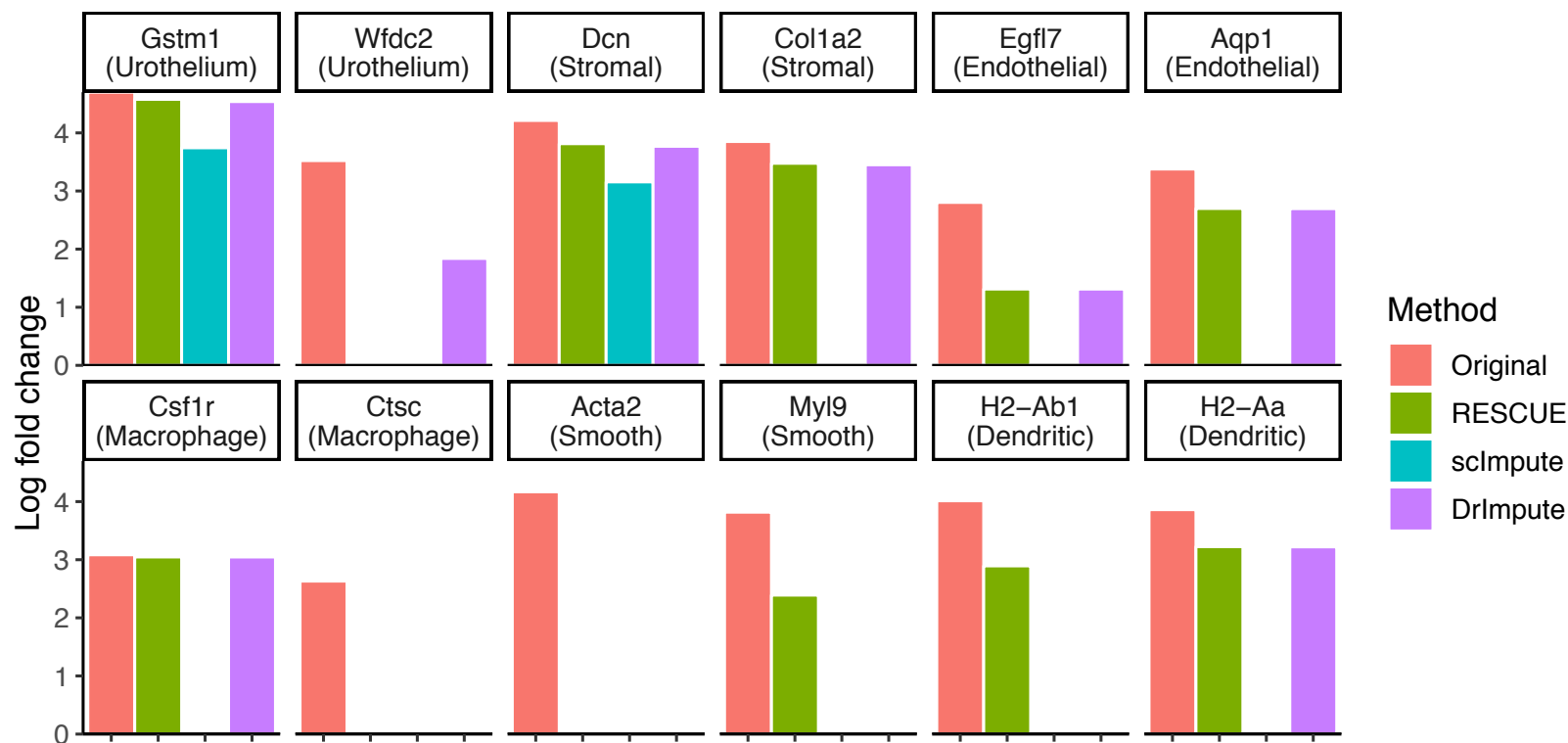

Supplement: Supplementary file 5 — Figure S5. Estimation bias after imputing the MCA bladder tissue data. (a) The percent absolute error for all missing counts. (b) The percent error for counts specific to top marker genes across cell types. Above 100% indicates no improvement over the data containing simulated dropout. (c) Log-fold changes in the two most differentially expressed marker genes for each cell type that went undetected after dropout. (PDF 67 kb) [file 12859_2019_2977_MOESM5_ESM.pdf]

**Fig. S6****a**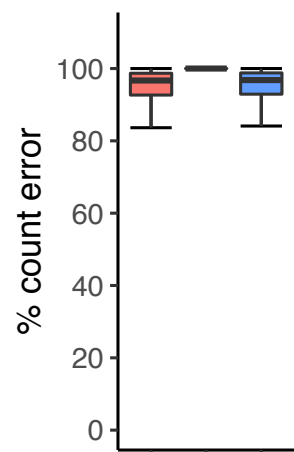**b**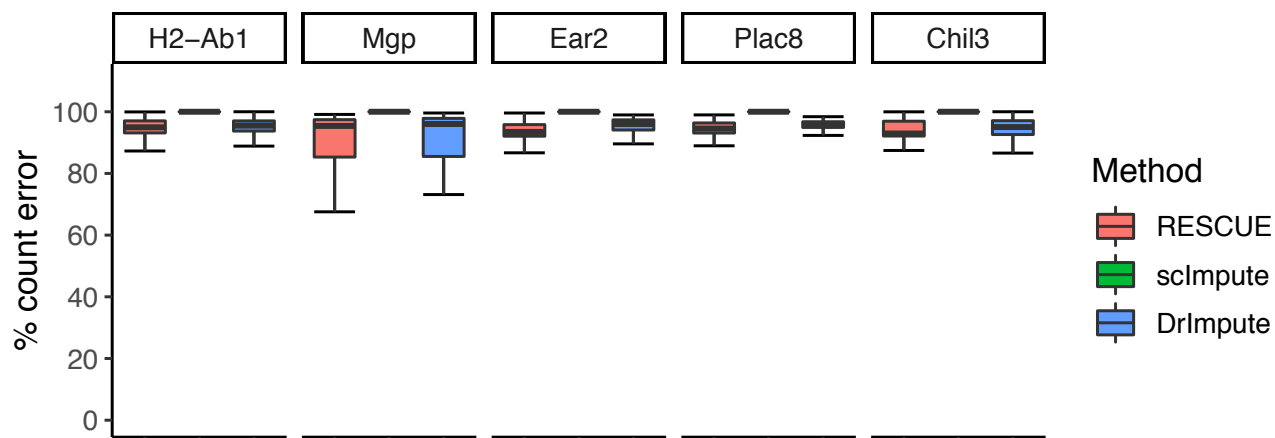**c**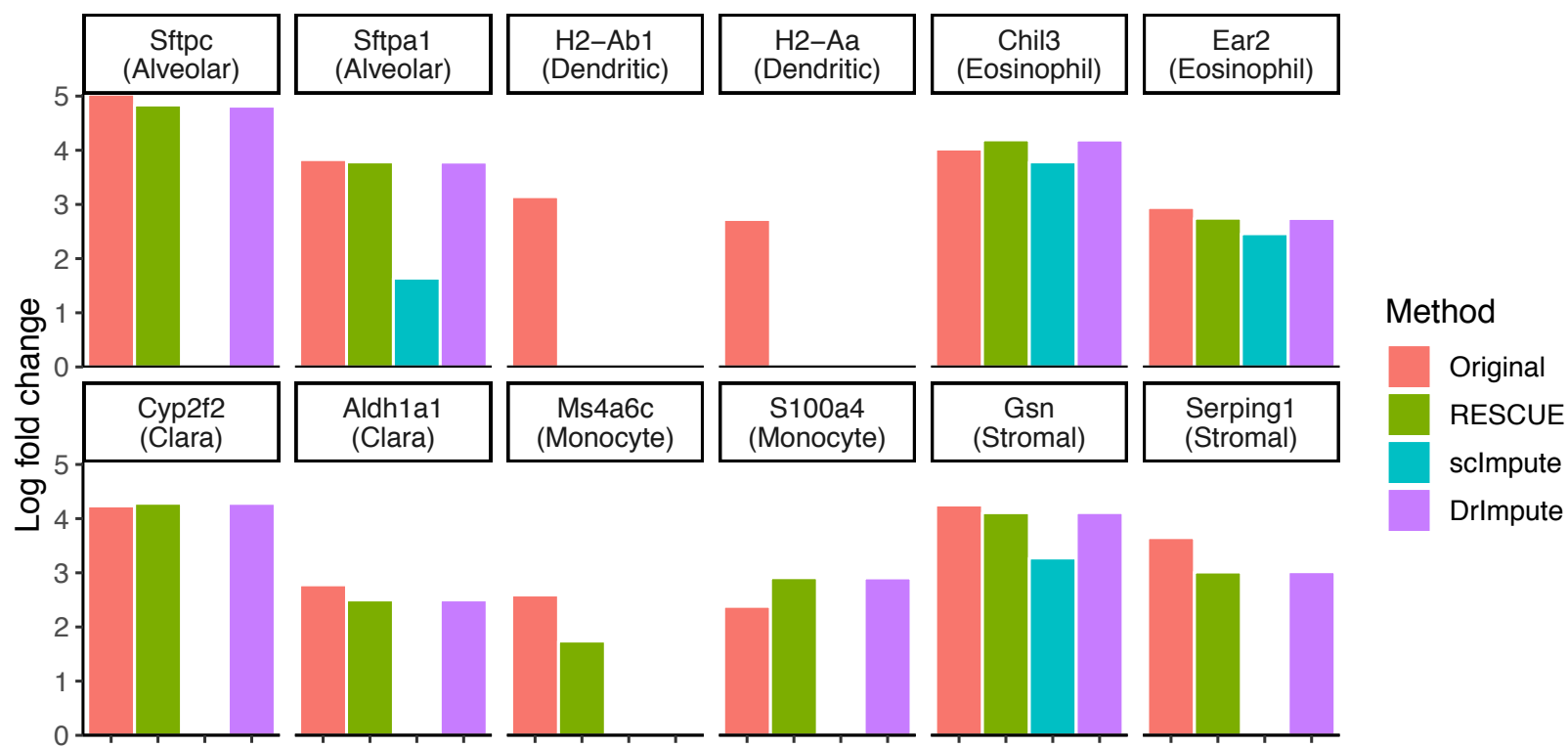

Supplement: Supplementary file 6 — Figure S6. Estimation bias after imputing the MCA lung tissue data. (a) The percent absolute error for all missing counts. (b) The percent error for counts specific to top marker genes across cell types. Above 100% indicates no improvement over the data containing simulated dropout. (c) Log-fold changes in the two most differentially expressed marker genes for each cell type that went undetected after dropout. (PDF 70 kb) [file 12859_2019_2977_MOESM6_ESM.pdf]

**Fig. S7****a**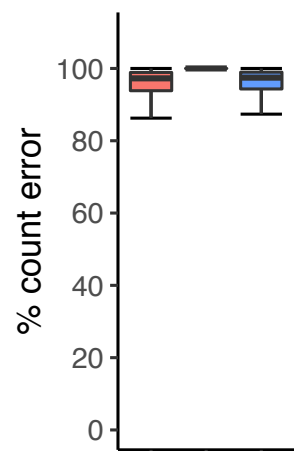**b**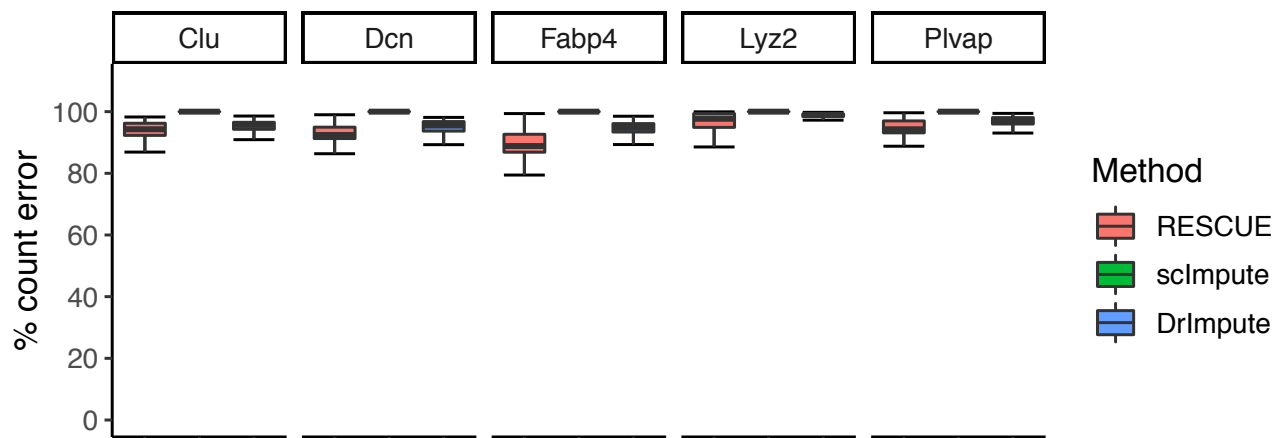**c**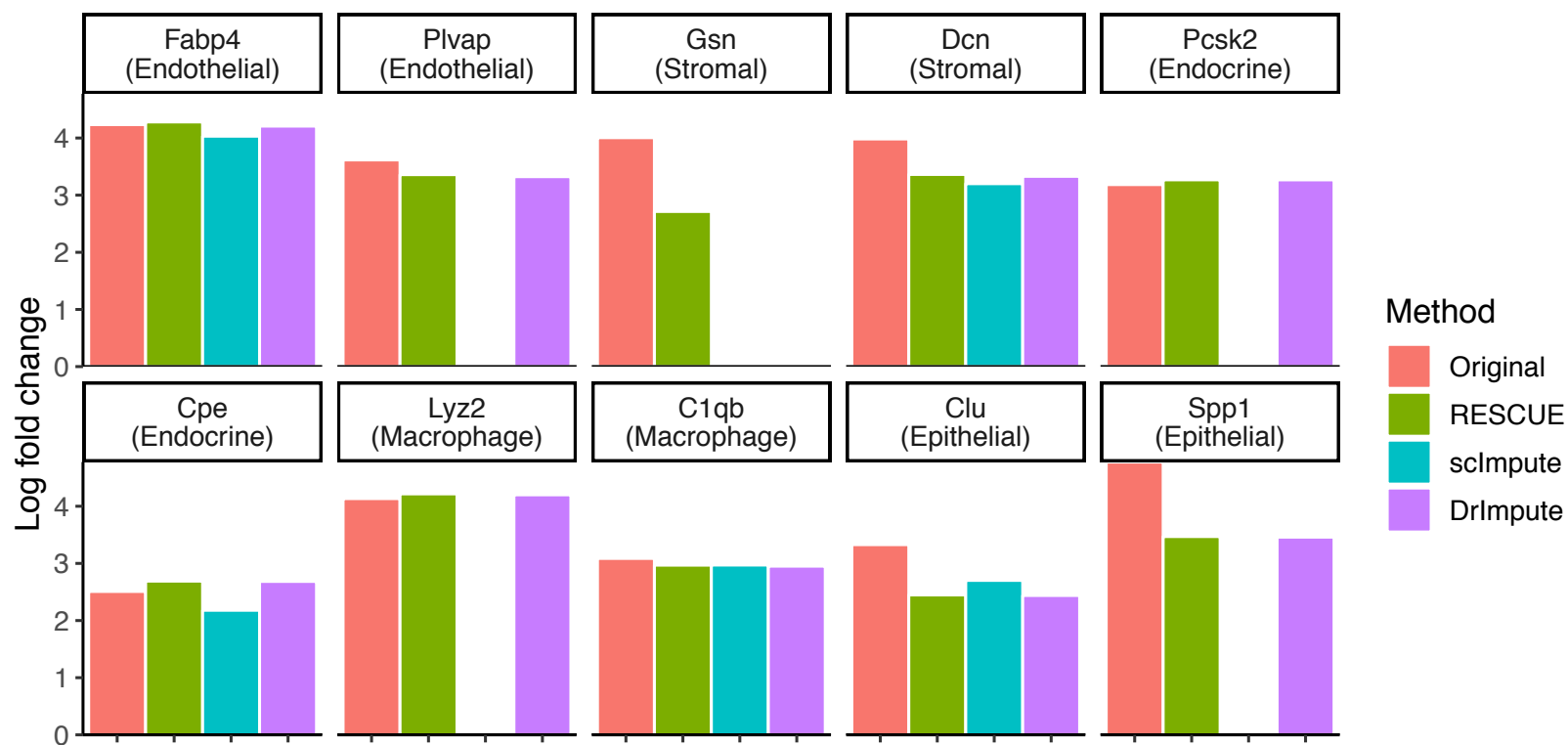

Supplement: Supplementary file 7 — Figure S7. Estimation bias after imputing the MCA pancreas tissue data. (a) The percent absolute error for all missing counts. (b) The percent error for counts specific to top marker genes across cell types. Above 100% indicates no improvement over the data containing simulated dropout. (c) Log-fold changes in the two most differentially expressed marker genes for each cell type that went undetected after dropout. (PDF 62 kb) [file 12859_2019_2977_MOESM7_ESM.pdf]

**Fig. S8**

**a**

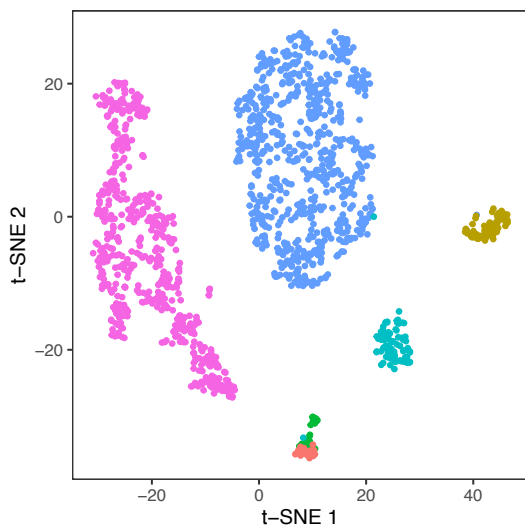

**b**

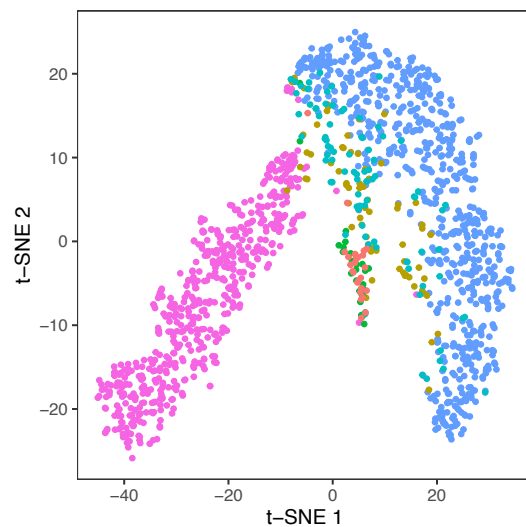

**c**

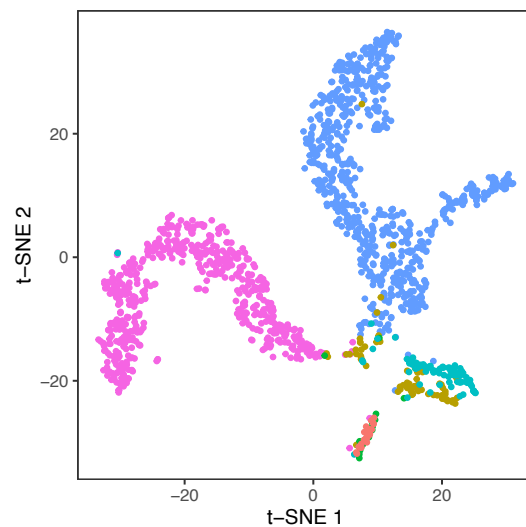

**d**

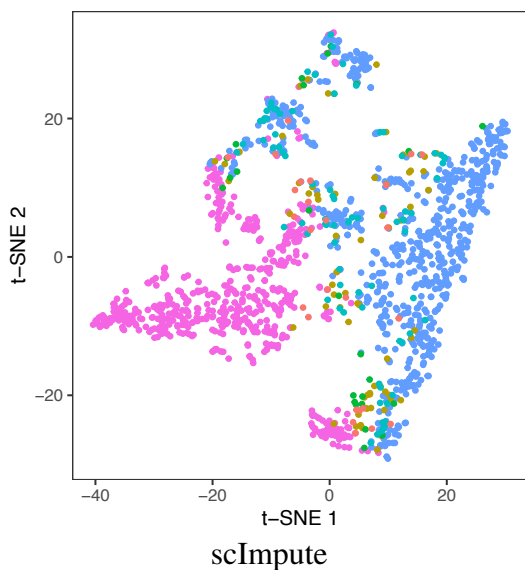

**e**

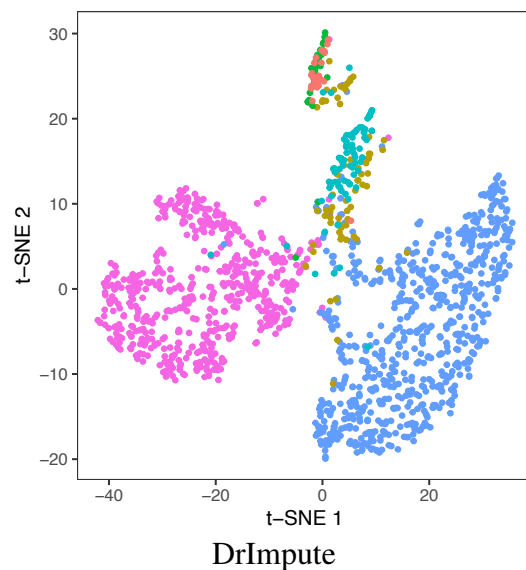

**f**

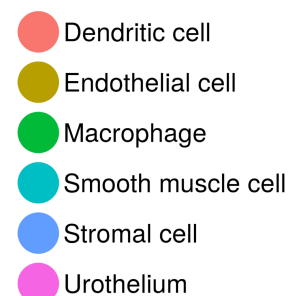

Supplement: Supplementary file 8 — Figure S8. Data visualization before and after imputing the MCA bladder tissue data. (a) t-SNE visualization of the original data labeled by cell type. (b) t-SNE after dropout (c) t-SNE after application of RESCUE. (d) t-SNE after application of scImpute. (e) t-SNE after application of DrImpute. (PDF 966 kb) [file 12859_2019_2977_MOESM8_ESM.pdf]

**Fig. S9**

**a**

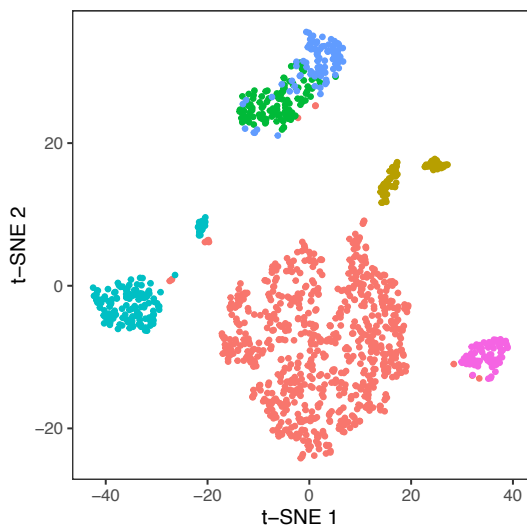

**b**

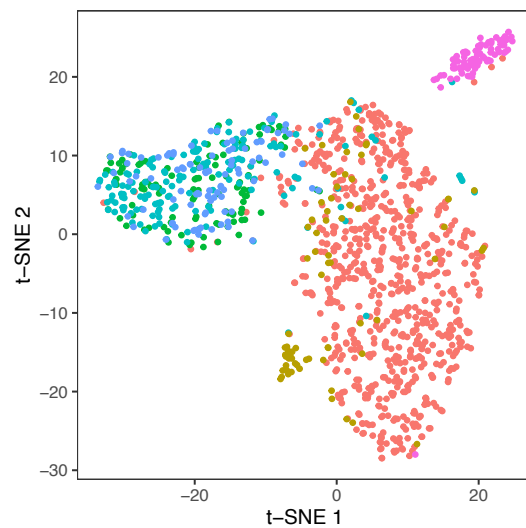

**c**

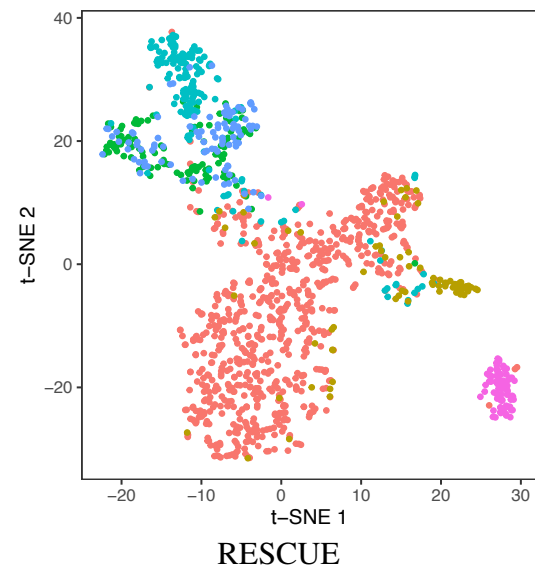

**d**

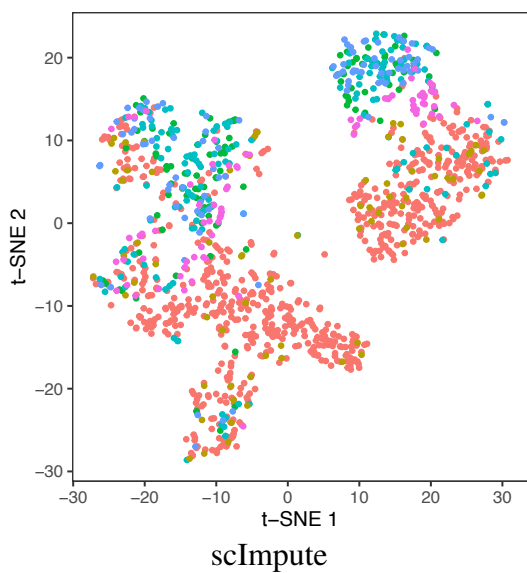

**e**

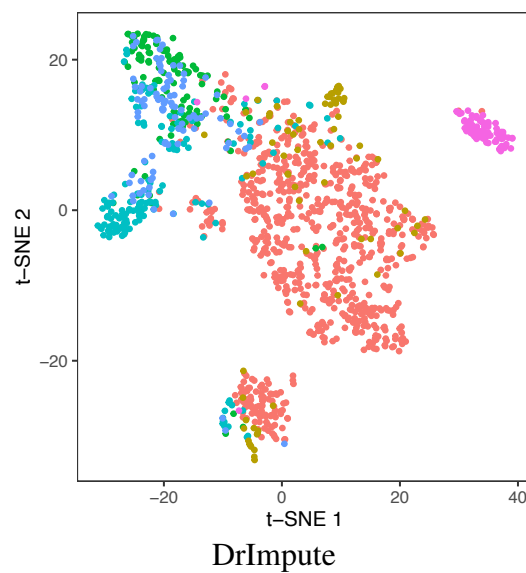

**f**

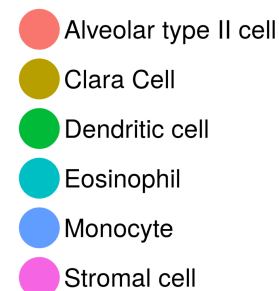

Supplement: Supplementary file 9 — Figure S9. Data visualization before and after imputing the MCA lung tissue data. (a) t-SNE visualization of the original data labeled by cell type. (b) t-SNE after dropout (c) t-SNE after application of RESCUE. (d) t-SNE after application of scImpute. (e) t-SNE after application of DrImpute. (PDF 888 kb) [file 12859_2019_2977_MOESM9_ESM.pdf]

**Fig. S10**

**a**

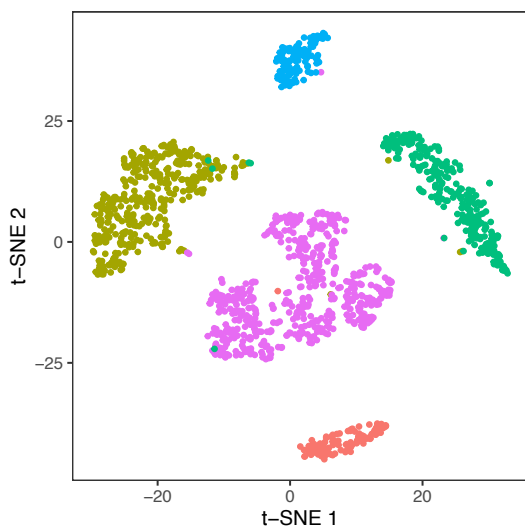

**b**

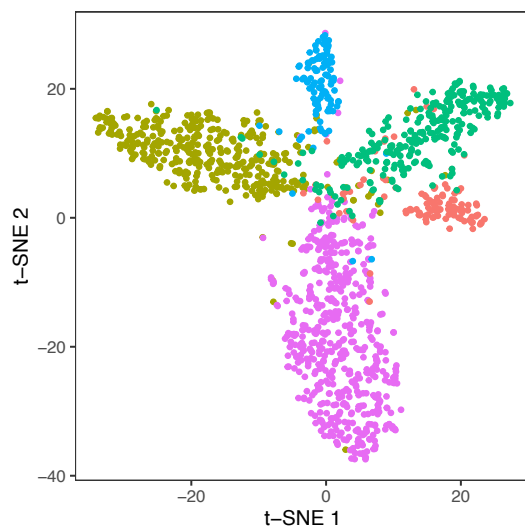

**c**

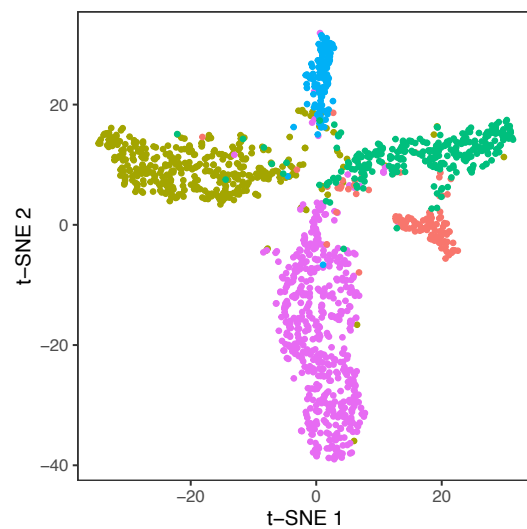

**d**

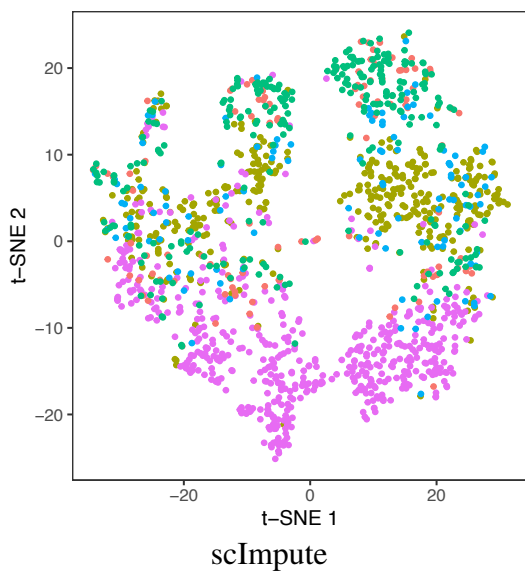

**e**

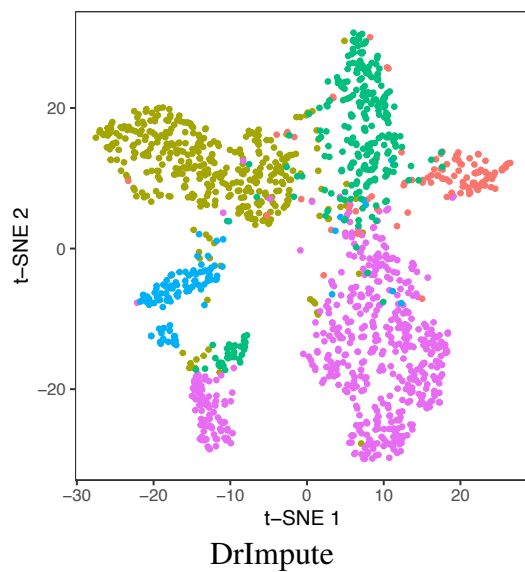

**f**

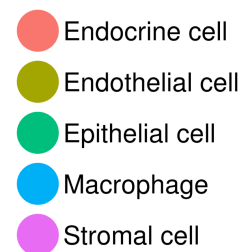

Supplement: Supplementary file 10 — Figure S10. Data visualization before and after imputing the MCA pancreas tissue data. (a) t-SNE visualization of the original data labeled by cell type. (b) t-SNE after dropout (c) t-SNE after application of RESCUE. (d) t-SNE after application of scImpute. (e) t-SNE after application of DrImpute. (PDF 917 kb) [file 12859_2019_2977_MOESM10_ESM.pdf]

**Fig. S11**

**a**

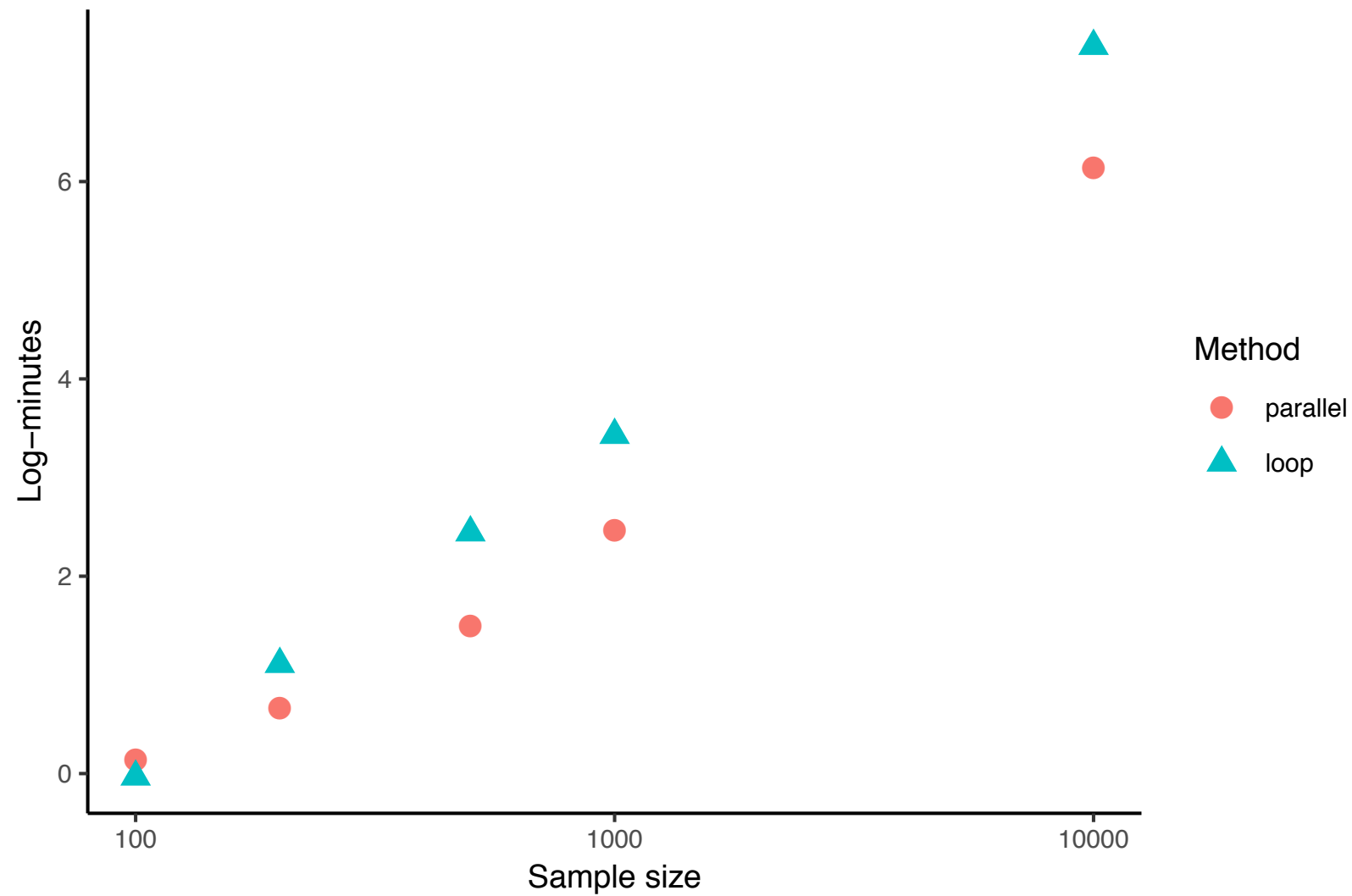

Supplement: Supplementary file 11 — Figure S11. Minutes of the RESCUE computation against sample size in Splatter simulations on the natural log-scale. (PDF 44 kb) [file 12859_2019_2977_MOESM11_ESM.pdf]

**Fig. S12**

**a**

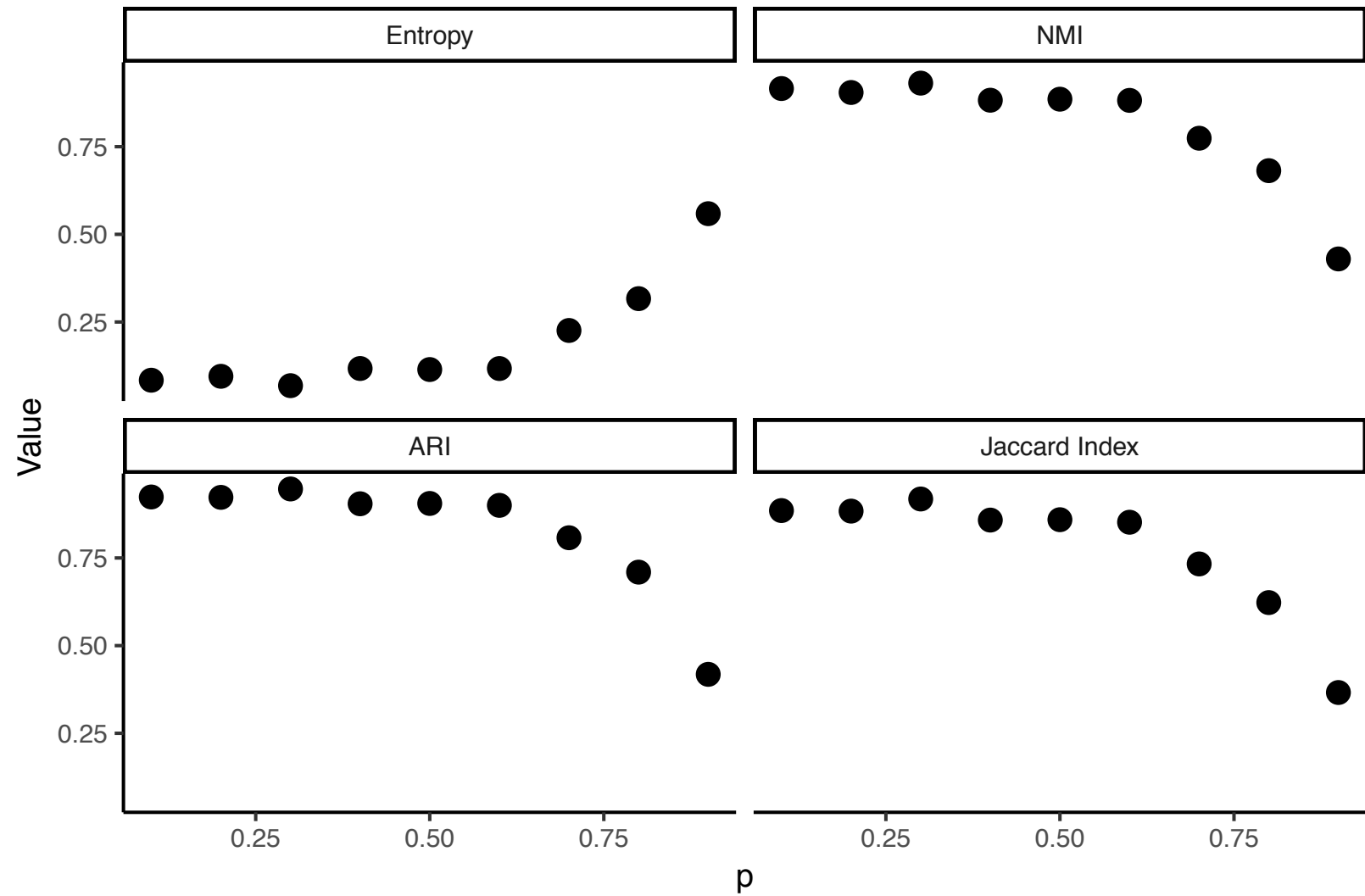

Supplement: Supplementary file 12 — Figure S12. Similarity measures between imputed and original data with different proportions p of subsampled genes in the first simulation scenario and the dropout rate parameter to − 0.25 in order to encourage the need for subsampling HVGs. (PDF 40 kb) [file 12859_2019_2977_MOESM12_ESM.pdf]

**Fig. S13**

**a**

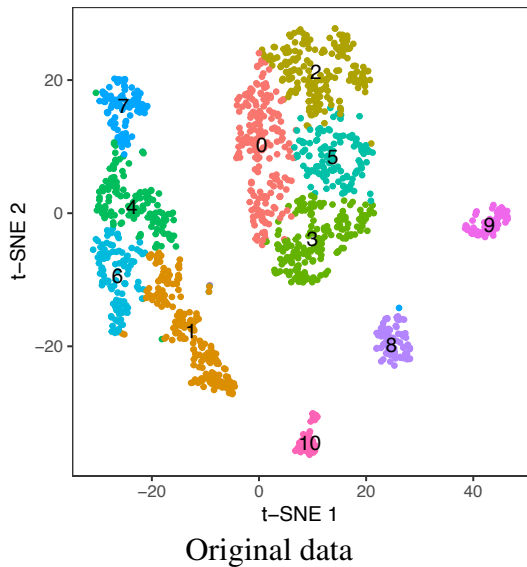

**b**

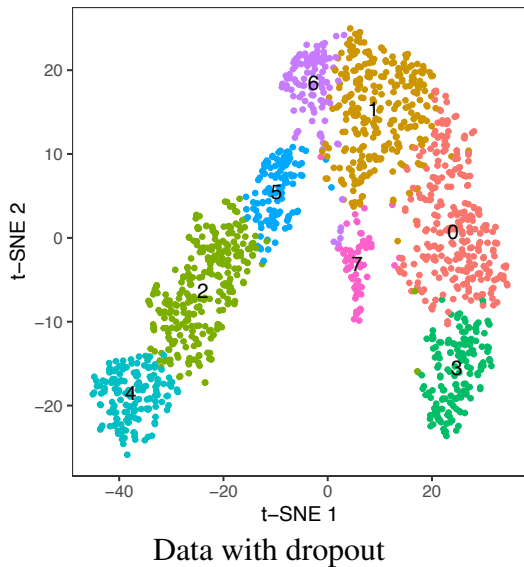

**c**

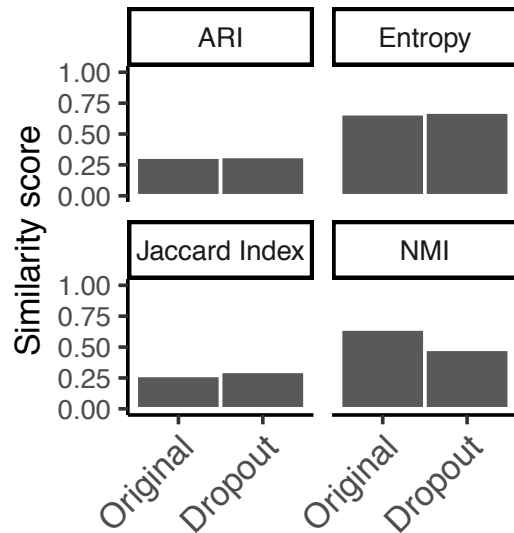

Supplement: Supplementary file 13 — Figure S13. Data visualization and clustering results before and after dropout in the MCA bladder tissue. (a) t-SNE visualization of the original uterus tissue data labeled by estimated clusters. (b) t-SNE after dropout. (c). (PDF 272 kb) [file 12859_2019_2977_MOESM13_ESM.pdf]
