# Supplementary material for: RESCUE: imputing dropout events in single-cell RNA-sequencing data
Source: BMC Bioinformatics. 2019 Jul 12;20:388. doi: 10.1186/s12859-019-2977-0 (PMC6624880; doi:10.1186/s12859-019-2977-0)
Supplement: Supplementary file 14 — Table S1. Splatter simulation parameters. (DOCX 14 kb) [file 12859_2019_2977_MOESM14_ESM.docx]

**Supplemental Table 1.** Splatter simulation parameters.

| Parameter | Data Scenario | | |
| --- | --- | --- | --- |
|  | Primary | Scenario 2 | Scenario 3 |
| nGenes | 10000 | 1000 | 10000 |
| batchCells | 500 | 501 | 500 |
| seed | 940 | 940 | 940 |
| group.prob | (0.2, 0.2, 0.2, 0.2, 0.2) | (0.1,0.3,0.6) | (0.2,0.4,0.4) |
| de.prob | 0.05 | 0.1 | 0.05 |
| dropout.mid | (0, 0, 0, 0, 0) | (0, 0, 0) | (0, 0, 0) |
| dropout.shape | (-0.5, -0.5, -0.5, -0.5, -0.5) | (-0.1, -0.1, -0.1) | (-0.25, -0.25, -0.25) |
