# Supplementary material for: RESCUE: imputing dropout events in single-cell RNA-sequencing data
Source: BMC Bioinformatics. 2019 Jul 12;20:388. doi: 10.1186/s12859-019-2977-0 (PMC6624880; doi:10.1186/s12859-019-2977-0)
Supplement: Supplementary file 15 — Table S2. Significant differentially expressed genes. (DOCX 14 kb) [file 12859_2019_2977_MOESM15_ESM.docx]

**Supplemental Table 2.** Significant differentially expressed genes (MAST likelihood ratio test *p* $<1e-5$; log-fold change $>2$).

| Number of genes | Method | Tissue | | | |
| --- | --- | --- | --- | --- | --- |
|  |  | Bladder | Lung | Pancreas | Uterus |
| Detected in original, lost to dropout | Original data | 123 | 87 | 106 | 77 |
|  | RESCUE | 86 | 60 | 85 | 53 |
|  | scImpute | 27 | 20 | 32 | 19 |
|  | DrImpute | 77 | 65 | 61 | 50 |
| Detected total | Original data | 171 | 119 | 133 | 100 |
|  | RESCUE | 172 | 143 | 133 | 111 |
|  | scImpute | 66 | 60 | 59 | 41 |
|  | DrImpute | 162 | 143 | 113 | 108 |
